# Genetic Dissection of Alzheimer’s Disease Using *Drosophila* Models

**DOI:** 10.3390/ijms21030884

**Published:** 2020-01-30

**Authors:** Youngjae Jeon, Jae Ha Lee, Byoungyun Choi, So-Yoon Won, Kyoung Sang Cho

**Affiliations:** 1 Department of Biological Sciences, Konkuk University, Seoul 05029, Korea; hhghhh@naver.com (Y.J.); aquari2@naver.com (J.H.L.); quddbs316@konkuk.ac.kr (B.C.); 2 Department of Biochemistry, Chungbuk National University College of Medicine, Cheongju 28644, Korea

**Keywords:** AD model, Alzheimer’s disease, amyloid β, *Drosophila*, functional genomics

## Abstract

Alzheimer’s disease (AD), a main cause of dementia, is the most common neurodegenerative disease that is related to abnormal accumulation of the amyloid β (Aβ) protein. Despite decades of intensive research, the mechanisms underlying AD remain elusive, and the only available treatment remains symptomatic. Molecular understanding of the pathogenesis and progression of AD is necessary to develop disease-modifying treatment. *Drosophila*, as the most advanced genetic model, has been used to explore the molecular mechanisms of AD in the last few decades. Here, we introduce *Drosophila* AD models based on human Aβ and summarize the results of their genetic dissection. We also discuss the utility of functional genomics using the *Drosophila* system in the search for AD-associated molecular mechanisms in the post-genomic era.

## 1. Introduction

### 1.1. Genetics of Alzheimer’s Disease

Alzheimer’s disease (AD) is a progressive neurological disorder that results in irreversible loss of neurons, particularly in the cortex and hippocampus. As of 2019, over 50 million people worldwide have AD or a related dementia [1]; which leads to death within three to nine years after diagnosis [2].

In the brain of an AD patient, amyloid beta (Aβ)-containing senile plaques and neurofibrillary tangles (NFTs), the aggregates of hyperphosphorylated tau protein, are observed, which are the main hallmarks of AD [3]. Therefore, Aβ, the cleaved form of amyloid precursor protein (APP), and tau, a microtubule-binding protein, have been suggested to be important causative molecules in the pathology of AD [4,5]. Aβ can aggregate to form flexible soluble oligomers that are toxic to nerve cells [6]. F urthermore, the accumulation of Aβ protein causes AD-associated events such as formation of NFT, cell loss, vascular damage, and dementia [4]. Based on the increased prevalence of early onset AD (EOAD) in Down syndrome patients with three copies of the *APP* gene and in people with the *APP* gain of function mutation that increases Aβ levels, Aβ has been thought to be a major cause of AD [7,8,9]. Supporting this idea, gain-of-function mutations of *presenilin 1/2*, a gene encoding the components of γ-secretase that processes APP to Aβ, which increased accumulation of Aβ, have been also associated with EOAD [9]. On the other hand, hyperphosphorylated tau, a microtubule-associated protein that stabilizes the microtubules, aggregates to form the NFT, resulting in neuronal degeneration [10]. Pathological tau aggregation is found in the brains of most AD patients, and the association between Aβ accumulation and tau phosphorylation has been reported [10]. Therefore, abnormal aggregation of tau protein is considered an important event in the pathogenesis of AD.

However, in the last decade, all clinical trials targeting Aβ or tau have failed, and the only available treatment remains symptomatic [11]. Therefore, despite decades of intensive research, the cause of AD remains elusive. In fact, many studies have demonstrated that AD is a complicated multifactorial disorder and may be affected by the combination of various genetic and environmental factors [12,13]. A twin study suggested that, depending on the model applied, the heritability of AD is 58% to 79% even though nongenetic risk factors also play an essential role [14]. Therefore, it is important to identify various genetic risk factors associated with AD for early detection and intervention. A series of genome-wide association studies (GWAS) have identified several genetic risk loci for late onset AD (*LOAD*), which seem to cluster in patterns that suggest immunity, lipid processing, and endocytosis as important causal biological processes [13]. In addition, the functional relevance of many AD-related factors has been demonstrated through functional genomic studies using genetic models, such as nematodes, fruit flies, and zebra fish, as well as mice [15].

### 1.2. Drosophila Models of Alzheimer’s Disease

**Figure 1:**
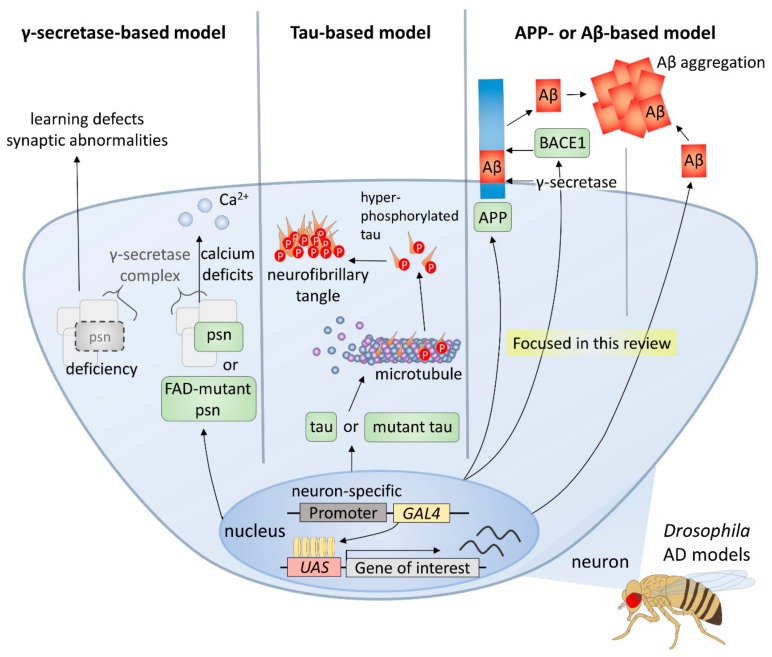
*Drosophila* models for Alzheimer’s disease.

Owing to the advantages of the *Drosophila* system, including the ability to withstand various genetic manipulations that cannot be performed in mammals, it has been an important model in AD studies [16,17] (Figure 1). There are three main types of *Drosophila* AD models according to the transgenes used, and the first type is the γ-secretase-based model. γ-secretase complex components are functionally conserved in the fly, and its many targets, such as APP and Notch receptor, are also conserved [18,19]. Overexpression of wild-type or familial AD-mutant *presenilin* (*psn*), a gene coding a component of γ-secretase complex, induces intracellular calcium deficits, which are regarded as one of the earliest events of AD pathology [20], whereas deficiency of *psn* causes associative learning defects and synaptic abnormalities in *Drosophila* larvae [21]. Thus, it follows, studies using γ-secretase-based AD models have facilitated understanding of the role of Presenilin in both development and degeneration as well as verifying many modifiers and pathways.

Furthermore, tau-based models have been established and used to study the role of tau in the formation of neurofibrillary tangles and neurotoxicity. For instance, several groups have shown that expression of human tau induces AD-like phenotypes in diverse *Drosophila* tissues [22,23]. A further study used a wild type or mutant human tau-expressing model to identify genetic modifiers of tau [24]. Moreover, the relationship between Aβ42 and tau has also been studied using Aβ42 and tau co-expression models [25].

Finally, most of the *Drosophila* AD models are based on APP or Aβ expression, since Aβ peptides, the major components of amyloid plaques, are considered to play the most important role in AD [26]. Because there is no conservation of both Aβ peptide sequence in APP and β-secretase in *Drosophila*, an essential condition for the generation of Aβ peptides, fly models expressing both human APP and BACE have been used [27,28,29]. In these models, AD-like phenotypes, such as age-dependent neuronal death, Aβ accumulation, and lethality are observed [27]. However, it has been found that APP-induced axonal defects are not caused by Aβ [30], and that the APP intracellular domain is involved in various processes such as axonal transport and synaptic plasticity [31]. Therefore, many groups have developed *Drosophila* AD models that directly express Aβ42 in the fly brain for a more direct study of the role of amyloid plaques in AD [32,33,34,35]. Each of the *UAS-Aβ42* transgenes produced by these groups have differences in some part of the construct, such as the signal peptide, poly A tail, or the number of *Aβ42* copies, which are directly related to the degree of Aβ peptide accumulation and intensity of AD-like phenotypes [36].

In this review, we will focus on the results obtained from models based on Aβ, the most commonly used AD models in *Drosophila*. The genetic modifiers found in studies using these *Drosophila* AD models to date suggest that several cellular pathways may be involved in the development of AD, and the results of these studies demonstrate the usefulness of the *Drosophila* model for finding related factors of multifactorial genetic diseases, such as AD.

## 2. AD-Related Mechanisms and Genetic Modifiers Identified Using the *Drosophila* Model

### 2.1. Amyloid Beta Accumulation

In the brain of *Drosophila* expressing *Aβ42*, age-dependent amyloid deposition was observed, as in human patient brains [37]. Moreover, ectopically expressed *Aβ42* in *Drosophila* photoreceptors showed amyloidogenic and aggregating properties; the resistance to proteolytic cleavage, increased structural stability, and toxicity [32,35,38,39,40].

Recently, several studies showed the role of templated protein misfolding, referred to as seeding [41,42], which induces misfolding and aggregation of the normal soluble protein [43]. Consistently, *Drosophila* models have provided evidence for a link between the seeding mechanism and neurotoxicity in vivo on a short time scale [44].

#### 2.1.1. Soluble Aβ Oligomer Toxicity and Aggregation

Soluble Aβ oligomer was observed in the CSF of human AD [45] and was more closely associated with disease severity than amyloid plaque, insoluble Aβ, or fibrillar species [46]. Moreover, in other studies using ELISA and Western blotting, the amount of soluble oligomer was found to be more decisive for cognitive deficits than the simple plaque counts [47], and these soluble peptides induced progressive neuronal loss [48]. Consistently, Aβ peptide generation in the *Drosophila* retina shows age-dependent neurodegeneration in retinal photoreceptor cells and precedes the formation of Aβ plaques, suggesting that the Aβ oligomer and protofibril mediate toxicity [27]. The structural importance of Aβ to generate oligomer is also proved in *Drosophila*. A flavonoid derivative that interferes and disorders the Aβ oligomer and inhibitors targeting the α-helix of Aβ prevented Aβ-induced neurotoxicity in a *Drosophila* transgenic AD model [49,50]. A study showed the genetic interaction of neuroserpin, a natural inhibitor of tissue-type plasminogen activator that forms a binary complex with Aβ and prevents mature fibril formation of Aβ, with Aβ ectopically expressed in vivo in the *Drosophila* AD model [51]. Moreover, recent studies have shown that the cytosolic and secreted forms of the heat shock protein 70 (HSP70) prevent Aβ42 self-aggregation by binding to Aβ42, in which this reduction in aggregation by HSP70 significantly improved the memory performance of flies expressing Aβ42 [52,53]. In contrast, the mammalian prion protein stabilized Aβ oligomers and enhanced Aβ neurotoxicity in *Drosophila* [54]. Meanwhile, a recent study suggested a new hypothesis of a Aβ aggregation mechanism that gangliosides are responsible for Aβ assembly, by showing that ectopic expression of ganglioside synthesis enzymes in *Drosophila*, such as β1,4-galactosyltransferases (*B4GalT6*) and α2,3-sialyltransferase (*SAT1*), accelerate Aβ assembly [55].

#### 2.1.2. Aβ Degradation

Since the accumulation of Aβ is critical in AD pathology, the Aβ catabolic pathway-related factors should be very important in the control of AD. The in vivo function of neprilysin (NEP) and insulin degradation enzyme (IDE) that is involved in the Aβ catabolic pathway were tested in the *Drosophila* AD model. NEP belongs to the Aβ degrading enzymes, inhibition of which resulted in the pathologic deposition of Aβ in rats [56]. In the fly model, neuronal expression of human *NEP* led to significantly reduced intraneuronal deposits of Aβ42 in the brain and also suppressed Aβ42-induced neuron loss, suggesting that up-regulation of neuronal NEP activity is protective against intraneuronal Aβ42 accumulation and neuron loss [57]. IDE, a thiol metalloendopeptidase that cleaves small proteins, including insulin, has Aβ degrading activity [58], and *IDE* loss-of-function mutant mice showed elevated levels of neuronally secreted Aβ [58]. Consistent with mammals, the reduced lifespan of flies expressing *APP* and *BACE* in neurons was partially recovered by *Drosophila Ide* or human *IDE* expression, suggesting that IDE can inhibit the pathological processes associated with Aβ accumulation in vivo [59]. More recently, a study showed that partial knockout of neuronal Src homology 2B1 (*SH2B1*), an adaptor protein that is important for insulin receptor signaling, increased Aβ42 accumulation and had a detrimental effect on *Aβ42*-expressing flies, while overexpression of neuronal *SH2B1* decreased Aβ42 accumulation and had beneficial effects [60]. These results suggested that the insulin signaling pathway plays important roles in Aβ metabolism.

The autophagic-lysosomal pathway is another important Aβ clearance pathway, the in vivo function of which in AD pathology is revealed in *Drosophila*. The hyperactivated PI3K/AKT/mTOR pathway, a negative-regulating pathway against autophagy, is linked to disrupted clearance of Aβ and tau [61] and alterations in this pathway are associated with autophagic dysfunction in the AD brain [62]. In *Drosophila*, genetic or pharmacologic inhibition of the PI3K/AKT/mTOR pathway improved Aβ-induced memory loss [63]. A recent study showed that ectopic expression of human Thioredoxin-80 (*Trx80*), a truncated form of Thioredoxin-1, prevents the toxic effects of Aβ and inhibits its aggregation [64]. In the same study, Gerenu and colleagues found that Trx80 exerts its protective activity through activation of autophagy. In addition, human phospholipase D3 (PLD3), a type-II transmembrane protein of the PLD family, exerts a neuroprotective effect against toxicity caused by Aβ when ectopically expressed in AD model flies, and the role of PLD3 in lysosome dynamics was considered to contribute to the beneficial effect of PLD3 [65]. Given the importance of autophagy in degenerative brain diseases, it is expected that more autophagy-related genes will be found as modifiers in AD pathology.

#### 2.1.3. Intraneuronal Accumulation of Aβ

In addition to extracellular deposition, intraneuronal accumulation of Aβ has been revealed to be involved in pathological features of AD such as synaptic deficits, amyloid plaque formation, and cell death [66,67]. In the brains of AD patients, oligomeric Aβ is mainly localized in neurons, where it associates with lipid membranes [68]. As phosphoinositides, such as PI and PI4,5P, facilitate Aβ assembly in/on lipid membranes [69], their metabolizing enzymes affect AD pathogenesis by influencing neuronal accumulation of Aβ. Supporting this idea, a reduction in synaptojanin-1, which converts PI4,5P into PI4P, inhibited synaptic and behavioral impairments in *APP* transgenic mice [70,71]. In *Drosophila*, the functions of the PI4KIIIα complex, which controls the levels of plasmalemmal PI4P and PI4,5P, are well conserved. Genetic reduction of components, such as PI4KIIIα, rolling blackout (*RBO*), tetratricopeptide repeat domain 7 (*TTC7*), and Hyccin, of the PI4KIIIα complex suppressed the phenotypes of *Aβ42*-expressing flies by reduction of neuronal Aβ accumulation [72,73]. In addition, another genetic modifier screening study identified *Drosophila* orthologues of human 4-hydroxyphenylpyruvate dioxygenase (*HPD*) and proline rich mitotic checkpoint control factor (*PRCC*) as suppressors of intraneuronal accumulation of Aβ [74]. Although HPD functions as 4-hydroxyphenylpyruvate dioxygenase that catalyzes the conversion of 4-hydroxyphenylpyruvate to homogentisate and PRCC may have a role in pre-mRNA splicing, the molecular mechanisms underlying how these proteins affect the intraneuronal accumulation of Aβ remain to be elucidated.

### 2.2. Amyloid-Mediated Tauopathy

Since phosphorylation of tau plays an important role in AD, there is a lot of interest in the regulators of tau phosphorylation. *Drosophila* has a tau homolog protein containing conserved disease-related phosphorylation sites of human tau [75]. Several studies have reported that Aβ42 induces phosphorylation and pathology of tau in flies and further noted that tau plays an important role in the downstream processes of Aβ-induced toxicity [25,76,77,78]. Aβ42 enhances tau-induced toxicity such as axonal transport defects, neuronal dysfunction, and reduced survival in Aβ42/tau co-expressing flies [25]. These effects also have been consistently observed in several studies using cell and rodent models [79,80,81,82,83,84]. In double-transgenic mice, hyperphosphorylated tau aggregation was decreased by clearance of Aβ, while Aβ accumulation was not affected by increasing tau [82].

Interestingly, it is known that par-1, a *Drosophila* orthologue of microtubule/microtubule-associated protein affinity regulating kinase (MARK), and glycogen synthase kinase 3β (GSK3β), a component of the Wnt pathway, are critical for Aβ-induced tau phosphorylation in the fly model [25,78]. Several in vitro studies support the idea that Aβ promotes tau phosphorylation via GSK3β activity [85,86,87,88], and studies using mouse models indicate that MARK phosphorylates tau [89,90]. In human cell models, more than 40 tau phosphorylation sites are associated with AD [91,92,93,94]: Serine (Ser) 262 and Ser356 are phosphorylated by MARK [89,90], and Threonine (Thr) 231, Ser199, Ser202, Ser235, Ser396, and Ser404 by GSK3β [95,96,97,98]. In *Drosophila*, par-1 and GSK3β can also phosphorylate most of these phosphorylation sites of tau [99], suggesting that the catalytic function of the two kinases is conserved between insects and humans. Furthermore, it was first observed in flies that phosphorylation of tau by par-1 plays an important role in the subsequent phosphorylation of tau by GSK3β, suggesting that tau phosphorylation happens in a structurally arranged pattern [99,100]. When Ser262 and Ser356 in tau are substituted for non-phosphorylatable Ala, Aβ42-mediated tau phosphorylation at Thr231 by GSK3β is blocked [78].

In mice, Aβ42 toxicity occurs through Aβ-induced phosphorylation of tau, and reduction or clearance of tau alleviates phenotypes and toxicity of Aβ42; for instance, memory impairment, synaptic loss, neuron loss, and premature death [83,101]. Furthermore, a study showed that removal of endogenous *Drosophila* tau reduces Aβ42-induced locomotor dysfunction in flies [102]. However, another recent study revealed that deletion of endogenous *Drosophila* tau had no effect on Aβ42-induced premature death in the fly model [103]. Therefore, unlike in mammals, it is controversial whether endogenous *Drosophila* tau contributes to Aβ42-induced toxicity.

### 2.3. Modifiers Related to Stress-Responsive Pathways

#### 2.3.1. Oxidative Stress

It has been known that Aβ42 causes oxidative stress, which is believed to be augmented in the brain of AD patients and animal models [104,105,106]. Based on the important role of oxidative stress in AD pathology, the interest in genes associated with oxidative stress has increased. Although several groups have shown that these genes affect AD pathology through screening studies in *Drosophila* [107,108,109,110,111], the effects of antioxidant genes, such as superoxide dismutase (*SOD*), on AD remain controversial.

Rival and colleagues carried out unbiased screening to identify genes whose expressions were changed by Aβ42 and found that antioxidative stress genes, such as Ferritin 1 heavy chain homolog (*Fer1HCH*), Ferritin 2 light chain homolog (*Fer2LCH*), catalase (*CAT*), and *Sod2*, reduced Aβ42-induced toxicity [107]. In other studies, *Fer1HCH* and *Fer2LCH* inhibited Aβ42-induced eye phenotype and premature death [108], while knockdown of *Fer1HCH* and *Fer2LCH* increased Aβ42-induced toxicity [109]. In addition, overexpression of *sarah* (*sra*), a *Drosophila* orthologue of protein-serine/threonine phosphatase regulator Down Syndrome Critical Region 1, increases the hydrogen peroxide susceptibility and enhances Aβ42-induced toxicity as a result of reducing the expression of *Sod2*, *Sod3*, and Glutathione S transferase D1 in *Aβ42*/*sra*-coexpressing flies [111].

In contrast, Favrin and colleagues identified 712 genes of whose expressions were changed in *Aβ42*-expressing flies and demonstrated that knockdown of *Sod3*, an extracellular superoxide dismutase gene whose expression increases in *Aβ42*-expressing flies, increases the locomotor defect and premature death in AD model flies [112]. Expression of wild type *Sod1*, another ROS-associated gene, decreases the lifespan of *Aβ42*-expressing flies, while a dominant negative form of Sod1 rescues premature death of the AD model flies [107]. These detrimental effects of Sods on *Aβ42*-expressing flies may be due to the toxic hydrogen peroxide overload, which occurs because of an imbalance between Sod, which produces hydrogen peroxide, and CAT, which catalyzes the decomposition of hydrogen peroxide to water and oxygen. Furthermore, the functional differences between these SODs may be also due to the distinct locations and prosthetic groups of the enzymes: Sod1 and Sod3 is Cu/ZnSOD in the cytoplasm and extracellular space, respectively, and Sod2 is MnSOD in the mitochondria [113,114]. Therefore, it is possible that there is a difference in ROS function between the mitochondria and cytoplasm in AD pathology.

It is believed that factors related with metals also play important roles in the cellular response to oxidative stress [115]. ROS generated by Aβ42 damages the cellular membrane, especially in the presence of metals such as copper, zinc, and iron [115]. Intake of copper or zinc enhances Aβ42 toxicity, while genetic inhibition of copper transporter 1B (Ctr1B) or Ctr1C, copper importers, or zinc/iron regulated transporter-related protein 1 (Zip1), a zinc importer, alleviates premature death and locomotor defects in AD model flies [116,117].

AD is strongly associated with oxidative stress, and many oxidative stress-related genes, including metals- and mitochondria-related genes, affect AD pathology. Although many antioxidant genes have been shown to have beneficial effects on AD, the protective effect of SOD remains controversial and further studies should be conducted.

#### 2.3.2. Endoplasmic Reticulum Stress

Endoplasmic reticulum (ER) stress has also been implicated in AD [118] and can be induced by Aβ42 in cultured cells, mice, and flies [35,119,120,121,122]. During the course of ER stress, a fragment of activating transcription factor 6 (ATF6) moves into the nucleus and expresses ER stress response genes, including X-box binding protein 1 (*XBP1*) [123]. Expression of *XBP1* is also promoted by Aβ42, and overexpression of spliced *XBP1* (*XBP1-S*), the activated form of XBP1, reduces Aβ42 toxicity in *Drosophila* photoreceptors, whereas knockdown of endogenous *XBP1* intensifies rough eye phenotype induced by Aβ42 [35]. Moreover, a recent study showed that overexpression of *XBP1-S* in the fly brain reduces Aβ42 levels and improves Aβ42-induced locomotor dysfunction, while reduction of endogenous *XBP1* increases Aβ42 protein levels and enhances Aβ42-induced locomotor dysfunction [124]. Furthermore, chronic expression of *Aβ42* activates protein kinase R-like endoplasmic reticulum kinase (PERK) and ATF6 pathways, both major branches of the unfolded protein response, as well as inositol-requiring enzyme 1 α-XBP1 pathway, and Aβ42-induced activation of PERK may have a beneficial effect on AD by Aβ42 clearance [124]. Therefore, *Drosophila* studies suggest that the ER stress response pathways may be implicated in the pathogenesis of AD.

### 2.4. Modifiers Involved in ERK Pathway or Cell Cycle

#### 2.4.1. EGFR/ERK Signaling

Activation of the extracellular signal-regulated kinase (ERK) pathway, as well as other mitogen-activated protein kinases (MAPKs) that include c-jun N-terminal kinase (JNK) and p38 MAPK, has been observed in AD neurons and animal models [125,126,127,128]. The ERK pathway has been reported to play a crucial role in dead signaling in neurons [129,130,131], although it is commonly thought to be a survival signal [132,133]. In *Drosophila*, Aβ42 activates ERK, and pharmacological inhibition of ERK activity reduces neurotoxicity of Aβ42, suggesting that chronic activation of ERK is a crucial step in the progression of AD [131]. Furthermore, extracts of Chinese medical herbs, such as *C. sativum* and *N. jatamansi* that inhibit ERK activation, ameliorate AD phenotypes in cultured mammalian cells and flies [134,135]. Epidermal growth factor receptor (EGFR) signaling within a particular range is necessary to maintain homeostasis of mushroom bodies, which is required for neuronal plasticity, learning, and memory [136]. However, excessive EGFR aggravates short-term memory loss of *Aβ42*-expressing flies, while treatment with gefitinib or erlotinib, two EGFR inhibitors, suppresses Aβ42-induced memory loss [137].

#### 2.4.2. Cell Cycle

Erroneous cell cycle re-entry (CCR) in neurons has been considered to be a crucial causative factor in neuronal death [138]. In AD brains, vulnerable neurons show activated cell cycle phenotypes, such as abnormally elevated cell cycle markers and re-expression of cell cycle regulators. Yet they are incapable of completing the cell cycle, resulting to the aberrant neuronal death [139,140]. The Aβ42 oligomer induces CCR in cultured primary neurons, and neuronal CCR takes place before accumulation of Aβ42 in *APP*-expressing rat brains [141,142]. Consistently, in the *Drosophila* brain, Aβ42 increases expression of Cyclin B, an important cell cycle protein, while genetic reduction of *Cyclin B* extends the lifespan and improves locomotor dysfunction of AD flies [143]. APP also induces erroneous CCR, and knockdown of *polo*, another key regulator of the cell cycle, partially rescues APP-induced locomotor dysfunction and retinal degeneration and prevents a shortened lifespan by repressing APP-induced CCR [144].

It is also known that Notch activation, which is essential for neuronal specification and development, is implicated in erroneous CCR and AD [145,146,147]. In the rodent model, kainic acid-induced activation of Notch results in neurodegeneration through erroneous CCR [146]. Moreover, genetic reduction of Delta, a ligand of Notch, and N-[N-(3,5-Difluorophen-acetyl)-Lalanyl]-S-phenylglycine t-butyl ester, a Notch inhibitor, rescued learning impairments and prevented the premature death of *Aβ42*-expressing flies [147].

### 2.5. Modifiers Related to Apoptosis

Apoptosis is a major pathway of neurodegenerative cell death in AD, and several apoptosis-related factors have been identified as genetic modifiers of AD pathology [23,148]. In *Drosophila*, apoptotic cell death induced by ectopic expression of *Aβ42* was detected by several methods, including active caspase 3 antibody staining, TUNEL assay, and acridine orange staining in the fly brain [53,149]. Moreover, several studies have shown that anti-apoptotic proteins have protective effects on AD-like phenotypes in *Aβ42-* or *tau*-expressing flies [24,38,150]. For example, co-overexpression of baculovirus p35, a caspase inhibitor, partially inhibits Aβ42-induced cell death in fly photoreceptors [38], implying that Aβ42-mediated cell death occurs via both p35-sensitive caspase-dependent and -independent pathways. In contrast, another study showed that death-associated inhibitor of apoptosis 1 (DIAP1), a *Drosophila* homolog of the inhibitor of apoptosis proteins, almost completely suppressed the AD-like phenotype, including Aβ42-induced cell death in the fly brain [150]. Given that Dronc, an initiator of caspase, can be inhibited by DIAP1 but not by p35 [151,152,153], the difference in the degree of protective effects between p35 and DIAP1 suggests the role of Dronc in Aβ42-induced cell death. In addition, deficiency of the translocator protein 18 kDa (*TSPO*), an outer mitochondrial membrane protein that plays an important role in the regulation of apoptosis, rescues the reduced lifespan of *Aβ42*-expressing flies by decreasing apoptosis and caspase 3 and 7 activities [154].

JNK and the stress-activated protein kinase subfamily have been also implicated in AD pathology [155,156,157,158]. JNK signaling is up-regulated by Aβ42 and also contributes to Aβ42-induced cell death in *Drosophila* [38,150]. Furthermore, inhibition of JNK through genetic modification or pharmacological treatment alleviates Aβ42-induced neuronal cell death, reduced survival rate, and locomotor dysfunction in flies [150]. Additionally, night-time sleep loss of *Aβ42*-expressing flies is restored by JNK inhibition [159].

The detrimental effect of JNK in AD model flies has been also revealed through the modulation of JNK upstream and downstream factors [24,150,160,161]. Hemipterous (hep), a *Drosophila* homolog of the JNK kinase, enhances Tau-induced toxicity in fly eyes [24], while *hep* deficiency reduces neuronal cell death of *Aβ42*-expressing larvae [160]. Additionally, a deficiency of *Drosophila* forkhead box subgroup O (*dFOXO*), a downstream factor of JNK, suppressed Aβ42-induced neuronal cell death, the reduced survival rate, and locomotor dysfunction of *Aβ42*-expressing flies [150]. Expression of human amyloid precursor like protein-1 (*APLP1*), a component of the amyloid precursor protein family, in flies increases transcription of dFOXO target pro-apoptotic genes, *hid* and *reaper*, and APLP1-induced cell death is rescued by genetic reduction of dFOXO [161].

A recent study has highlighted another aspect of neuronal cell death in the brain of AD model flies [162]. Apoptosis promotes the elimination of impaired neurons from brain circuits, protecting the brain instead of attacking it. In the same study, Coelho and colleagues demonstrated that suppression of fitness-based removal of Aβ42-damaged neurons by knockdown of *azot*, the fitness checkpoint gene, and overexpression of *DIAP1* exacerbated AD-like phenotypes of *Aβ42*-expressing flies, including degenerative vacuoles, a decreased lifespan, a locomotory defect, and a memory defect. This new finding shows that the role of neuronal cell death in the AD brain is more complex than previously thought.

### 2.6. Modifiers Related to Epigenetic Regulation

Based on its important function in neurons, epigenetic mechanisms have been suggested to play a pivotal role in AD pathophysiology [163]. Histone acetylation, which is regulated by the activities of histone deacetylases (HDAC) and histone acetyltransferases (HAT), has been primarily implicated in AD-like phenotypes in *Drosophila*. HDAC6 is a unique member of the HDAC family that acts mainly on cytoplasmic non-histone substrates [164] and increased in a postmortem study of human AD brain [165]. A study of mammals showed that Aβ-induced mitochondrial transport was rescued by inhibition of HDAC6 [166]. In *Drosophila*, microtubule defects in human *tau*-expressing flies were rescued by HDAC6 inhibition [167]. However, another study indicated that histone deacetylase inhibitor trichostatin A caused lethality and delayed development in *Drosophila* [168], as well as inducing neuronal death in mice [169].

γ-cleavage of APP releases an intracellular tail that forms a complex with Tip60, a member of the HAT family, which affects the pathophysiology of AD [170]. In *APP*-overexpressing transgenic mice, levels of Tip60 are increased [171]. In contrast, in *Drosophila*, Tip60 levels are decreased, while HDAC1 levels are increased, in the larval brain of *APP*-overexpressing flies, which resulted in epigenetic repression of neuroplasticity genes [172]. Moreover, specific loss of Tip60 activity enhanced APP-mediated lethality and neuronal apoptotic cell death in *Drosophila*, while overexpression of *Tip60* diminished these defects and improved the learning and memory performance of *APP*-expressing larvae [172,173]. Another HAT family protein, the CREB-binding protein (CBP), has also been reported to play a neuroprotective role in the *Drosophila* AD model. The expression of CBP in *Drosophila* expressing *Aβ42* in the retina rescued eye phenotype, apoptosis, neurodegeneration, and axonal targeting defects. This neuroprotective effect of CBP was found to be essential for the bromo, HAT, and poly glutamine stretch (BHQ) domain of CBP [174].

### 2.7. Modifiers Related With Synaptic Abnormalities

Failure of normal synaptic function might be one of the earliest measurable deficits in AD [175], and the decreases in synaptic density appear to occur early in the course of AD in mouse models and patients [176,177,178,179].

Aβ peptides have been suggested to have physiological roles in synaptic function [180], and Aβ oligomers specifically target molecular components that mediate synaptic plasticity [181]. In several studies, mutant *APP* transgenic mice showed synaptic dysfunction before plaque formation, suggesting that soluble Aβ levels in the cortex significantly correlate with the degree of synaptic loss [178,182,183,184]. Studies in *Drosophila* have shown that early memory defects and structural and/or functional synaptic defects are induced by expression of *Aβ*, and that Aβ peptides inhibit the formation and/or maturation of new synapses [37,63,185,186]. Thus, antibodies to neutralize soluble Aβ oligomers are suggested as treatment for early AD [187]. Consistent with these findings, various factors that act on normal synaptic function have been found to be AD-related factors, as follows.

#### 2.7.1. Small GTPases

Small GTPases, such as Rho and Rac1, play a prominent role in the development of dendritic structure [188]. For example, the constitutive active (CA) form of RhoA reduces dendritic arbor growth in *Drosophila* [189], and CA Rac1 tends to form ruffle-like structure in rodent neurons [190].

Several studies implicated these small GTPases in AD pathology. It has been shown that *RhoA* expression was decreased in synapses and increased in dystrophic neurites in a mouse AD model, suggesting that RhoA might be associated with AD pathology [191]. In *Drosophila*, increased activation of Rho1, the *Drosophila* orthologue of vertebrate RhoA, by prenylation, a posttranslational modification facilitating the association of proteins with membranes, leads to age-dependent degeneration of the nervous system [192]. A recent study demonstrated that Rac1 activity was abnormally increased in the hippocampal tissues of AD patients and mouse AD models, and that inhibition of the elevated Rac1 activity rescued memory loss in both fly and mouse AD models [193].

#### 2.7.2. Impaired Axonal Transport

Impaired transportation in neurons is regarded as an underlying cause of synaptic failure in AD [194], and overexpression of *APP* leads to axonal transport defects in fly and mouse models [195,196,197]. In the peripheral nerves of *Drosophila* larvae expressing *APP*, a “traffic jam” of vesicles was observed, and expression of scaffolding proteins, Fe65 and JIP1b, which interact with the APP intracellular domain, also induced the axonal transport defect [198].

#### 2.7.3. Synaptic Proteins

It has been shown that the expression levels of genes involved in synaptic vesicle trafficking are decreased in the brain of AD patients [199]. In flies, expression of Bruchpilot, a homolog of ELKS/RAB6-interacting/CAST family member 2 (*ERC2*) that can affect the localization of Ca^+2^ channels in presynaptic release sites [200], was not significantly different in *APP*-and *BACE*-expressing larvae compared to the control group [201] and five-day-old *Aβ*-expressing flies, but showed reduced levels at 21 days of age [202]. Additionally, the levels of Discs-large, a homolog of *DLG4* essential for the density of the synaptic glutamate receptor [203,204], were significantly reduced in fly larvae expressing *APP* and *BACE* [201]. A recent study proved the importance of the synaptic localization of a subset of synaptic proteins, including APP [205]. In this study, Furotani and colleagues demonstrated that knockdown of *yata*, a novel gene regulating the synaptic localization of β amyloid protein precursor-like and other synaptic proteins, rescued the phenotypes of *APP*-expressing flies, suggesting that the regulators of the synaptic localization of synaptic proteins are involved in AD pathology.

### 2.8. Mitochondrial Dysfunction or Mislocation

Mitochondrial dysfunction has been reported to contribute to the progression of AD pathology [206]. For example, Dynamin-related protein 1 (Drp1), a key regulator of mitochondrial fission, exerts a beneficial effect in *Aβ42*-expressing flies by protecting mitochondria [207]. In contrast, *sra* overexpression reduced the number of mitochondria and enhanced Aβ42 toxicity [111]. Also, the mislocation of mitochondria has been implicated in AD pathology. Mitochondria are dynamic organelles whose active movement is essential for the mobilization of the synaptic vesicle reserve pool [208]. In the brains of AD patients and the AD model mouse, disruption of mitochondrial transport resulted in the reduced distribution of mitochondria in the axon and dendrites [196]. Furthermore, mislocalization of mitochondria was observed in the brain of flies expressing *Aβ42* [209]. Therefore, restoration of mitochondrial transportation could be a promising target mechanism that can be used to develop AD therapies.

### 2.9. Modifiers Related with Inflammation

The immune system has been known to be aberrantly activated in the brains of AD patients and contributes to AD pathogenesis [210,211]. As the immune response not only provides a protective role by promoting the clearance of toxic Aβ aggregates but also exerts a detrimental effect on AD pathology by up-regulating chronic inflammation, it has been regarded as a double-edged sword in neurodegenerative disease [212]. In particular, innate immunity and neuroinflammation are thought to be essential components of neurodegeneration in both fly and mammalian brains [213,214,215].

#### 2.9.1. Phagocytic Receptor Draper

After Aβ is produced in the CNS, binding of soluble Aβ oligomer and fibrils to the microglial receptor produces a signal that results in the production of cytokines and chemokines [216]. In *Drosophila*, glial cells show functional and morphological similarity to those of mammals [217] and contribute to the protection of neurons, engulfing Aβ fibrils by phagocytosis in the fly AD model [218]. Glial phagocytosis may decrease with aging due to decreased levels of Draper, a *Drosophila* homolog of the mammalian Multiple EGF Like Domains 10, which is a conserved phagocytic receptor of glial cells [219], and up-regulation of Draper which can reverse the Aβ-related phenotype [218], suggesting that the phagocytic function of glial cells may exert a beneficial effect in neurodegenerative disease.

#### 2.9.2. TREM2

Triggering Receptor Expressed on Myeloid cell 2 (TREM2) is another receptor that mediates phagocytosis on the microglial surface [220] and regulates inflammatory responses via Toll-like receptors (TLRs) in AD [221,222]. TREM2 suppresses inflammation through the inhibition of cytokine production [223] and has a protective effect in AD by reducing inflammation-induced neuronal damage [224]. TREM2 mutation correlates with a significantly increased risk of AD [225], and TREM2 deficiency promotes Aβ accumulation due to a dysfunctional microglial response [226]. There is no apparent *Drosophila* homolog of *TREM2*. However, a recent study using *Drosophila* AD models shows that glial expression of human *TREM2* or human tyrosine kinase binding protein (*TYROBP*), the intracellular adaptor of TREM2, did not affect AD-like phenotypes of *Aβ42*-expressing flies, while glial expression of *TREM2*/*TYROBP* modifies molecular signatures induced by neuronal expression of Aβ42 [227]. Unlike Aβ42, *TREM2*/*TYROBP* expression in glial cells exacerbated tau-mediated AD pathology [227]. Therefore, further detailed research using various AD models, on the functions of TREM2 in AD pathology is needed.

#### 2.9.3. Toll and IMD Pathways

Toll and immune deficiency (IMD) pathways are the major innate immune responsive pathways in *Drosophila* that regulate the production of antimicrobial peptides [228]. The Toll pathway that is mainly responsive to fungi, Gram-positive bacteria, and virulence factors, functions through the NF-kB family transcription factors, Dorsal and Dif, while the IMD pathway, responsive to Gram-negative bacteria, acts through another NF-kB family transcription factor, Relish.

In mammals, several TLRs are involved in Aβ uptake by microglial cells and activate innate immune responses to prevent Aβ accumulation in the CNS [221,229,230]. In AD brains, high expression of *TLR* was detected [231], and *TLR*-deficient mice showed increased Aβ deposits [221], suggesting that TLR is involved in Aβ clearance. However, loss-of-function mutations of *Drosophila* Toll (*Tl*) suppresses the pathological effects of human Aβ42 in the *Drosophila* AD model [232]. Moreover, the same study showed that deficiencies in the downstream components of the Toll pathway, including an adaptor protein Tube, the IRAK-like kinase Pelle, and Dorsal and Dif, ameliorated the rough eye phenotype of human *Aβ42*-expressing flies [232]. The difference between the mammalian and fruit fly results probably reflects the dual aspect of the Toll pathway, i.e., clearance and inflammation.

The IMD pathway has also been implicated in the toxicity of Aβ42 in *Drosophila*. The expression of peptidogylcan recognition protein SC1b (*PGRP-SC1b*), a suppressor of the IMD pathway, is up-regulated in *Aβ42*-expressing flies, and knockdown of *PGRP-SC1b* ameliorated the reduced lifespan and locomotory defect of the fly AD model [112], suggesting that the IMD pathway is protective against Aβ42 toxicity. In contrast, a recent study showed that the downregulation of Relish, a downstream transcription factor of the IMD pathway, in *Drosophila* astrocytes ameliorated the toxicity of Aβ42 as well as polyglutamine [233]. These confusing findings highlight the complexity of the role of the immune response in pathogenesis of AD.

## 3. Conclusions and Perspectives

**Figure 2:**
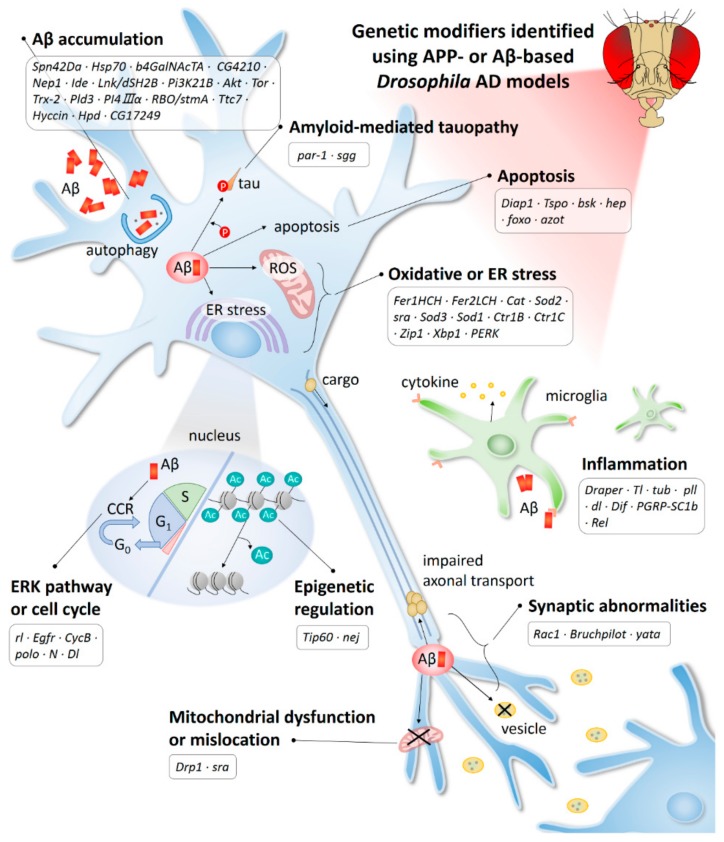
AD-related mechanisms identified using the APP-or Aβ-based *Drosophila* model. Ac, acetylation; CCR, cell cycle re-entry; ER, endoplasmic reticulum; ROS, reactive oxygen species.

In this study, we have reviewed the AD-associated genes found in the *Drosophila* models and their cellular pathways (Table 1 and Figure 2). Because AD is a multifactorial disease, various AD-related genetic factors and their functions need to be identified before developing accurate tests and treatments for this disease. Therefore, genetic studies focusing on the discovery of various AD-related genes are considered essential for identifying the causes of AD and developing therapies. With the recent application of GWAS and next-generation sequencing, 29 LOAD-related factors have been discovered, and research into the functions of these factors will broaden our understanding of AD etiology [234,235]. However, despite intensive research over the past decades, our understanding of the AD-related genes discovered to date is limited. In fact, a study showed that known AD-risk variants can explain only about 30% of AD variance, indicating that many genetic loci remain to be discovered [236]. Therefore, new approaches may be needed to boost the probability of identifying causal genes and pathways.

Functional genomic studies using *Drosophila* could be an alternative approach to finding new AD-related genes. The results of studies with *Drosophila* AD models described in this study are sufficient to show the feasibility of using *Drosophila* models to find new disease modifiers. While there are drawbacks—the *Drosophila* models are over simplified and have relatively low relevance to humans compared to mice—the advantages of using *Drosophila* models outweigh these drawbacks. Specifically, the *Drosophila* models have a wide variety of genetic tools, and it takes less than 10 days for the AD phenotype to appear, whereas it takes more than six months in the mouse AD model, although it varies from model to model [237], and allows for large-scale screening. In addition, the presence of RNAi for all genes annotated in its genome and simple crosses allow the screening of genetic modifiers for the toxicity of Aβ42 and tau. Therefore, more AD-related genes are expected to be discovered in the future thanks to *Drosophila* models. In particular, if there are limitations in the discovery of disease-related genes because of the small sample size in human GWAS studies, it will be possible to discover new genes by conducting a combined study of GWAS and *Drosophila* screening. *Drosophila* models will also be a good tool for in vivo functional studies of the genes discovered via human genetic studies. In the future, this combination of human genetics and *Drosophila* functional genomics is expected to be an important strategy for research into multifactorial diseases, such as AD.

In addition, *Drosophila* can also be used as a screening tool for drugs to modify AD symptoms or disease progression. Unfortunately, all recently developed AD treatment candidates have failed in the clinic and thus, the causative factors for the disease have come into question [238,239]. Despite such factors, AD progression, including accumulation of Aβ in the brain, begins long before the appearance of clinical symptoms such as memory loss, and most AD patients are diagnosed at a later stage of neurodegeneration. Recently, the FDA recognized this characteristic and issued an amendment to permit clinical trials very early in the disease [240]. As a result, it is anticipated that clinical trials of new drugs for these early diagnosis and early stage patients will increase rapidly. Therefore, finding more drug candidates will be another important task in this field.

*Drosophila* is a well-characterized insect with various phenotypes and is a model system that can be used to rapidly screen many drug candidates in vivo. Indeed, many studies to date have used the *Drosophila* AD model to screen for small molecules that can modify AD symptoms or validate the in vivo efficacy of developed drug candidates [29,241,242,243]. In addition, *Drosophila* has been used to screen natural products and traditional medicines that are expected to have therapeutic and prophylactic properties in AD [244,245]. In the future, the superiority of the *Drosophila* model as an in vivo drug screening system is expected to increase its utility.

## Figures and Tables

**Table 1:** Genetic modifiers of amyloid precursor protein (APP) or Aβ-based *Drosophila* Alzheimer’s disease (AD) models.

Pathway	*Drosophila* Genes	Human Genes	Protein Type and Function	Effect	References
Aβ production and aggregation	*Spn42Da*	*SERPINI*	Serine protease inhibitor that interacts with tissue plasminogen activator	S ^1^	[51]
	*Hsp70*	*HSPA1A*	Central component of the cellular network of molecular chaperones and folding catalysts	S	[52,53]
	*b4GalNAcTA*	*B4GALT6*	β-1,4-galactosyltransferase	E ^2^	[55]
	*CG4210*	*SAT1*	α-2,3-sialyltransferase	E	[55]
	*Nep1*	*MME*	Membrane metalloendopeptidase Aβ degrading enzyme	S	[57]
	*Ide*	*IDE*	Zinc metallopeptidase that degrades insulin	S	[59]
	*Lnk*/*dSH2B*	*SH2B1*	SH2-domain containing mediator important for insulin receptor signaling	S	[60]
	*Pi3K21B*	*PIK3R3*	Regulatory subunit of PI3K that phosphorylates phosphatidylinositol	E	[63]
	*Akt*	*AKT*	AKT serine/threonine kinase	E	[63]
	*Tor*	*MTOR*	Phosphatidylinositol kinase-related kinase	E	[63]
	*Trx-2*	*TXN*	Thioredoxin-1 that is involved in many redox reaction	S	[64]
	*Pld3*	*PLD3*	Enzyme that catalyzes the hydrolysis of membrane phospholipids	S	[65]
	*PI4KIIIα*	*PI4KA*	Lipid kinase that synthesizes phosphatidylinositol 4-phosphate from phosphatidylinositol	E	[72]
	*RBO/stmA*	*EFR3B*	Component of complex required to localize PI4K to the plasma membrane	E	[72]
	*Ttc7*	*TTC7B*	Component of complex required to localize PI4K to the plasma membrane	E	[73]
	*Hyccin*	*FAM126A*	Component of complex required to localize PI4K to the plasma membrane	E	[73]
	*Hpd*	*HPD*	4-hydroxyphenylpyruvate dioxygenase that catalyzes the conversion of 4-hydroxyphenyl-pyruvate to homogentisate	S	[74]
	*CG17249*	*PRCC*	Protein that may play a role in pre-mRNA splicing	S	[74]
Tauopathy	*par-1*	*MARK*	Serine/threonine kinase that plays critical roles in cell polarity and microtubule dynamics	E	[99]
	*sgg*	*GSK3β*	Serine/threonine kinase that plays multiple roles in various signaling pathways	E	[99]
Oxidative stress	*Fer1HCH*	*FTH1*	Subunit of Ferritin, an iron-storage protein	S	[107,108,109]
	*Fer2LCH*	*FTMT*	Subunit of mitochondrial Ferritin, an iron-storage protein	S	[107,108,109]
	*Cat*	*CAT*	Enzyme that catalyzes decomposition of hydrogen peroxide to water and oxygen	S	[107]
	*Sod2*	*SOD2*	Mitochondrial Mn-dependent superoxide dismutase	S	[107] [110]
	*sra*	*RCAN1*	Inhibitor of calcineurin	E	[111]
	*Sod3*	*SOD3*	Extracellular Cu/Zn-dependent superoxide dismutase	E	[112]
	*Sod1*	*SOD1*	Cytoplasmic Cu/Zn-dependent superoxide dismutase	E	[107]
	*Ctr1B*	*SLC31A1*	Copper importer	E	[117]
	*Ctr1C*	*SLC31A1*	Copper importer	E	[117]
	*Zip1*	*SLC39A3*	Zinc importer	E	[116]
ER stress	*Xbp1*	*XBP1*	Transcriptional factor that mediates the unfolded protein response	S	[35,124]
	*PERK*	*EIF2AK3*	ER transmembrane kinase that phosphorylates eukaryotic translation-initiation factor 2-alpha during ER stress	S	[124]
EGFR/ERK pathway	*rl*	*MAPK1*	Serine/threonine kinase and core component of the EGFR/MAPK pathway	E	[131]
	*Egfr*	*EGFR*	Membrane-localized tyrosine kinase receptor for epidermal growth factor	E	[137]
Cell cycle	*CycB*	*CCNB1*	G2/mitotic-specific protein which is a member of the cyclin family	E	[143]
	*polo*	*PLK1*	Serine/threonine kinase involved in mitosis	E	[144]
	*N*	*NOTCH1*	Transmembrane receptor for Notch signaling	E	[147]
	*Dl*	*DLL1*	Transmembrane ligand for Notch signaling	E	[147]
Apoptosis	*Diap1*	*BIRC2*	E3 ligase with inhibitory activity on caspase	S E	[150] [162]
	*Tspo*	*TSPO*	Outer mitochondrial membrane protein related to steroid and heme biosynthesis, apoptosis, protein import, cell proliferation, and differentiation	E	[154]
	*bsk*	*MAPK8*	Serine/threonine kinase that phosphorylates the Jra transcription factor	E	[38] [150]
	*hep*	*MAP2K7*	Serine/threonine kinase involved in the JNK pathway by phosphorylating JNK	E	[160]
	*foxo*	*FOXO*	Forkhead family of transcription factor regulated by various signaling pathway	E	[150] [161]
	*azot*	*CALM1*	EF-hand calcium binding protein act as fitness checkpoint	S	[162]
Epigenetic regulation	*Tip60*	*KAT5*	Histone acetyltransferase	S	[172]
	*nej*	*CBP*	Histone acetyltransferase	S	[174]
Synaptic abnormalities	*Rac1*	*RAC1*	GTPase which belongs to the RAS superfamily of small GTP-binding proteins	E	[193]
	*Bruchpilot*	*ERC2*	Cytoskeletal protein critical for structural integrity of electron-dense projection at pre-active zones	S	[202]
	*yata*	*SCYL1*	Transcriptional regulator belonging to the SCY1-like family of kinase-like proteins	E	[205]
Mitochondrial dysfunction or mislocation	*Drp1*	*DNM1L*	Dynamin related protein that regulates mitochondrial fission	S	[207]
	*sra*	*RCAN1*	Inhibitor of calcineurin	S E	[197] [111]
Inflammation and innate immune system	*Draper*	*MEFG10*	Multiple EGF like domains 10, which encodes a phagocytic receptor of glia	S	[218]
	*Tl*	*TLR*	Toll-like receptor that promotes NF-kB like transcription factors	E	[232]
	*tub*	*IRAK1*	Downstream component of the Toll pathway	E	[232]
	*pll*	*IRAK4*	Downstream component of the Toll pathway	E	[232]
	*dl*	*RELA*	Transcription factor regulated by the Toll pathway	E	[232]
	*Dif*	*RELB*	Transcription factor regulated by the Toll pathway	E	[232]
	*PGRP-SC1b*	*PGLYRP1*	Peptidoglycan recognition protein SC1b which is a negative regulator of Imd pathway	E	[112]
	*Rel*	*NFKB1*	Subunit 1 of nuclear factor kappa B, which is a main regulatory gene in the Imd pathway	E	[233]

^1^ Suppressor; ^2^ Enhancer.

## Abbreviations

AD	Alzheimer’s disease
Aβ	Amyloid β
APLP1	Amyloid precursor like protein 1
APP	Amyloid precursor protein
ATF6	Activating transcription factor 6
B4GALT6	β1,4-galactosyltransferases
BHQ	Bromo, HAT and poly glutamine stretch
CA	Constitutive active
CAT	Catalase
CCR	Cell cycle reentry
CBP	CREB-binding protein
Ctr1B	Copper transporter 1B
dFOXO	*Drosophila* forkhead box subgroup O
DIAP1	Death-associated inhibitor of apoptosis 1
Drp1	Dynamin-related protein 1
EGFR	Epidermal growth factor receptor
EOAD	Early onset AD
ER	Endoplasmic reticulum
ERC2	ELKS/RAB6-interacting/CAST family member 2
ERK	Extracellular signal-regulated kinase
Fer1HCH	Ferritin 1 heavy chain homologue
Fer2LCH	Ferritin 2 light chain homologue
GSK3β	Glycogen synthase kinase 3β
GWAS	Genome-wide association studies
HAT	Histone acetyltransferases
HDAC	Histone deacetylases
hep	hemipterous
HPD	4-hydroxyphenylpyruvate dioxygenase
Hsp70	Heat shock protein 70
IDE	Insulin degradation enzyme
IMD	Immune deficiency
JNK	c-jun N-terminal kinase
LOAD	Late onset AD
MARK	Microtubule affinity regulating kinase
MAPKs	Mitogen-activated protein kinases
NEP	Neprilysin
NFTs	Neurofibrillary tangles
PERK	Protein kinase R-like endoplasmic reticulum kinase
PGRP-SC1b	Peptidogylcan recognition protein SC1b
PLD3	Phospholipase D3
PRCC	Proline rich mitotic checkpoint control factor
psn	presenilin
RBO	Rolling blackout
SAT1	α2,3-sialyltransferase
Ser	Serine
SH2B1	Src homology 2B1
SOD	Superoxide dismutase
sra	sarah
Thr	Threonine
TLRs	Toll-like receptors
TREM2	Triggering receptor expressed on myeloid cell 2
Trx80	Thioredoxin-80
TSPO	Translocator protein 18 kDa
TTC7	Tetratricopeptide repeat domain 7
XBP1	X-box binding protein 1
XBP1-S	spliced *XBP1*
Zip1	zinc/iron regulated transporter-related protein 1

## References

[1] Alzheimer’s Disease International (2019). Executive summary. World Alzheimer Report 2019: Attitudes to Dementia.

[2] Cummings J.L. (2004). Alzheimer’s disease. N. Engl. J. Med..

[3] Ballard C., Gauthier S., Corbett A., Brayne C., Aarsland D., Jones E. (2011). Alzheimer’s disease. Lancet.

[4] Hardy J.A., Higgins G.A. (1992). Alzheimer’s disease: The amyloid cascade hypothesis. Science.

[5] Mudher A., Lovestone S. (2002). Alzheimer’s disease–do tauists and baptists finally shake hands?. Trends Neurosci..

[6] Haass C., Selkoe D.J. (2007). Soluble protein oligomers in neurodegeneration: Lessons from the Alzheimer’s amyloid β-peptide. Nat. Rev. Mol. Cell Biol..

[7] Wisniewski K., Wisniewski H., Wen G. (1985). Occurrence of neuropathological changes and dementia of Alzheimer’s disease in Down’s syndrome. Ann. Neurol..

[8] Mullan M., Crawford F., Axelman K., Houlden H., Lilius L., Winblad B., Lannfelt L. (1992). A pathogenic mutation for probable Alzheimer’s disease in the APP gene at the N–terminus of β–amyloid. Nat. Genet..

[9] Hardy J. (1997). Amyloid, the presenilins and Alzheimer’s disease. Trends Neurosci..

[10] Ballatore C., Lee V.M.-Y., Trojanowski J.Q. (2007). Tau-mediated neurodegeneration in Alzheimer’s disease and related disorders. Nat. Rev. Neurosci..

[11] Mehta D., Jackson R., Paul G., Shi J., Sabbagh M. (2017). Why do trials for Alzheimer’s disease drugs keep failing? A discontinued drug perspective for 2010-2015. Expert Opin. Investig. Drugs.

[12] Barnes D.E., Yaffe K. (2011). The projected effect of risk factor reduction on Alzheimer’s disease prevalence. Lancet Neurol..

[13] Bettens K., Sleegers K., Van Broeckhoven C. (2013). Genetic insights in Alzheimer’s disease. Lancet Neurol..

[14] Gatz M., Reynolds C.A., Fratiglioni L., Johansson B., Mortimer J.A., Berg S., Fiske A., Pedersen N.L. (2006). Role of genes and environments for explaining Alzheimer disease. Arch. Gen. Psychiatry.

[15] Van Dam D., De Deyn P.P. (2011). Animal models in the drug discovery pipeline for Alzheimer’s disease. Br. J. Pharmacol..

[16] Tan F.H.P., Azzam G. (2017). *Drosophila melanogaster*: Deciphering Alzheimer’s Disease. Malays. J. Med. Sci..

[17] Yeates C.J., Sarkar A., Kango-Singh M., Singh A., Mutsuddi M., Mukherjee A. (2019). Unraveling Alzheimer’s Disease Using *Drosophila*. Insights into Human Neurodegeneration: Lessons Learnt from Drosophila.

[18] Guo Y., Livne-Bar I., Zhou L., Boulianne G.L. (1999). *Drosophila* presenilin is required for neuronal differentiation and affects notch subcellular localization and signaling. J. Neurosci..

[19] Ye Y., Fortini M.E. (1999). Apoptotic activities of wild-type and Alzheimer’s disease-related mutant presenilins in *Drosophila melanogaster*. J. Cell Biol..

[20] Michno K., Knight D., Campusano J.M., van de Hoef D., Boulianne G.L. (2009). Intracellular calcium deficits in *Drosophila* cholinergic neurons expressing wild type or FAD-mutant presenilin. PLoS ONE.

[21] Knight D., Iliadi K., Charlton M.P., Atwood H.L., Boulianne G.L. (2007). Presynaptic plasticity and associative learning are impaired in a *Drosophila* presenilin null mutant. Dev. Neurobiol..

[22] Wittmann C.W., Wszolek M.F., Shulman J.M., Salvaterra P.M., Lewis J., Hutton M., Feany M.B. (2001). Tauopathy in *Drosophila*: Neurodegeneration without neurofibrillary tangles. Science.

[23] Jackson G.R., Wiedau-Pazos M., Sang T.K., Wagle N., Brown C.A., Massachi S., Geschwind D.H. (2002). Human wild-type tau interacts with wingless pathway components and produces neurofibrillary pathology in *Drosophila*. Neuron.

[24] Shulman J.M., Feany M.B. (2003). Genetic modifiers of tauopathy in *Drosophila*. Genetics.

[25] Folwell J., Cowan C.M., Ubhi K.K., Shiabh H., Newman T.A., Shepherd D., Mudher A. (2010). Aβ exacerbates the neuronal dysfunction caused by human tau expression in a *Drosophila* model of Alzheimer’s disease. Exp. Neurol..

[26] Tanzi R.E., Bertram L. (2005). Twenty years of the Alzheimer’s disease amyloid hypothesis: A genetic perspective. Cell.

[27] Greeve I., Kretzschmar D., Tschape J.A., Beyn A., Brellinger C., Schweizer M., Nitsch R.M., Reifegerste R. (2004). Age-dependent neurodegeneration and Alzheimer-amyloid plaque formation in transgenic *Drosophila*. J. Neurosci..

[28] Carmine-Simmen K., Proctor T., Tschape J., Poeck B., Triphan T., Strauss R., Kretzschmar D. (2009). Neurotoxic effects induced by the *Drosophila* amyloid-beta peptide suggest a conserved toxic function. Neurobiol. Dis..

[29] Chakraborty R., Vepuri V., Mhatre S.D., Paddock B.E., Miller S., Michelson S.J., Delvadia R., Desai A., Vinokur M., Melicharek D.J. (2011). Characterization of a *Drosophila* Alzheimer’s disease model: Pharmacological rescue of cognitive defects. PLoS ONE.

[30] Stokin G.B., Almenar-Queralt A., Gunawardena S., Rodrigues E.M., Falzone T., Kim J., Lillo C., Mount S.L., Roberts E.A., McGowan E. (2008). Amyloid precursor protein-induced axonopathies are independent of amyloid-beta peptides. Hum. Mol. Genet..

[31] Muller T., Meyer H.E., Egensperger R., Marcus K. (2008). The amyloid precursor protein intracellular domain (AICD) as modulator of gene expression, apoptosis, and cytoskeletal dynamics-relevance for Alzheimer’s disease. Prog. Neurobiol..

[32] Finelli A., Kelkar A., Song H.J., Yang H., Konsolaki M. (2004). A model for studying Alzheimer’s Abeta42-induced toxicity in *Drosophila melanogaster*. Mol. Cell. Neurosci..

[33] Crowther D.C., Kinghorn K.J., Miranda E., Page R., Curry J.A., Duthie F.A., Gubb D.C., Lomas D.A. (2005). Intraneuronal Abeta, non-amyloid aggregates and neurodegeneration in a *Drosophila* model of Alzheimer’s disease. Neuroscience.

[34] Iijima K., Iijima-Ando K. (2008). *Drosophila* models of Alzheimer’s amyloidosis: The challenge of dissecting the complex mechanisms of toxicity of amyloid-β 42. J. Alzheimers Dis..

[35] Casas-Tinto S., Zhang Y., Sanchez-Garcia J., Gomez-Velazquez M., Rincon-Limas D.E., Fernandez-Funez P. (2011). The ER stress factor XBP1s prevents amyloid-beta neurotoxicity. Hum. Mol. Genet..

[36] Jeon Y., Lee S., Shin M., Lee J.H., Suh Y.S., Hwang S., Yun H.S., Cho K.S. (2017). Phenotypic differences between *Drosophila* Alzheimer’s disease models expressing human Aβ42 in the developing eye and brain. Anim. Cells Syst..

[37] Iijima K., Chiang H.C., Hearn S.A., Hakker I., Gatt A., Shenton C., Granger L., Leung A., Iijima-Ando K., Zhong Y. (2008). Abeta42 mutants with different aggregation profiles induce distinct pathologies in *Drosophila*. PLoS ONE.

[38] Tare M., Modi R.M., Nainaparampil J.J., Puli O.R., Bedi S., Fernandez-Funez P., Kango-Singh M., Singh A. (2011). Activation of JNK signaling mediates amyloid-ss-dependent cell death. PLoS ONE.

[39] Moran M.T., Tare M., Kango-Singh M., Singh A. (2013). Homeotic Gene teashirt (tsh) has a neuroprotective function in amyloid-beta 42 mediated neurodegeneration. PLoS ONE.

[40] Steffensmeier A.M., Tare M., Puli O.R., Modi R., Nainaparampil J., Kango-Singh M., Singh A. (2013). Novel neuroprotective function of apical-basal polarity gene crumbs in amyloid beta 42 (aβ42) mediated neurodegeneration. PLoS ONE.

[41] Jucker M., Walker L.C. (2013). Self-propagation of pathogenic protein aggregates in neurodegenerative diseases. Nature.

[42] Jaunmuktane Z., Mead S., Ellis M., Wadsworth J.D., Nicoll A.J., Kenny J., Launchbury F., Linehan J., Richard-Loendt A., Walker A.S. (2015). Evidence for human transmission of amyloid-β pathology and cerebral amyloid angiopathy. Nature.

[43] Eisele Y.S. (2013). From Soluble A β to Progressive A β Aggregation: Could prion-like templated misfolding play a role?. Brain Pathol..

[44] Sowade R.F., Jahn T.R. (2017). Seed-induced acceleration of amyloid-β mediated neurotoxicity *in vivo*. Nat. Commun..

[45] Pitschke M., Prior R., Haupt M., Riesner D. (1998). Detection of single amyloid β-protein aggregates in the cerebrospinal fluid of Alzheimer’s patients by fluorescence correlation spectroscopy. Nat. Med..

[46] McLean C.A., Cherny R.A., Fraser F.W., Fuller S.J., Smith M.J., Vbeyreuther K., Bush A.I., Masters C.L. (1999). Soluble pool of Aβ amyloid as a determinant of severity of neurodegeneration in Alzheimer’s disease. Ann. Neurol..

[47] Näslund J., Haroutunian V., Mohs R., Davis K.L., Davies P., Greengard P., Buxbaum J.D. (2000). Correlation between elevated levels of amyloid β-peptide in the brain and cognitive decline. JAMA.

[48] Hardy J., Selkoe D.J. (2002). The amyloid hypothesis of Alzheimer’s disease: Progress and problems on the road to therapeutics. Science.

[49] Kumar A., Srivastava S., Tripathi S., Singh S.K., Srikrishna S., Sharma A. (2016). Molecular insight into amyloid oligomer destabilizing mechanism of flavonoid derivative 2-(4‘-benzyloxyphenyl)-3-hydroxy-chromen-4-one through docking and molecular dynamics simulations. J. Biomol. Struct. Dyn..

[50] Nerelius C., Sandegren A., Sargsyan H., Raunak R., Leijonmarck H., Chatterjee U., Fisahn A., Imarisio S., Lomas D., Crowther D. (2009). α-Helix targeting reduces amyloid-β peptide toxicity. Proc. Natl. Acad. Sci. USA.

[51] Kinghorn K.J., Crowther D.C., Sharp L.K., Nerelius C., Davis R.L., Chang H.T., Green C., Gubb D.C., Johansson J., Lomas D.A. (2006). Neuroserpin binds Aβ and is a neuroprotective component of amyloid plaques in Alzheimer disease. J. Biol. Chem..

[52] Martín-Peña A., Rincón-Limas D.E., Fernandez-Fúnez P. (2018). Engineered Hsp70 chaperones prevent Aβ42-induced memory impairments in a *Drosophila* model of Alzheimer’s disease. Sci. Rep..

[53] Fernandez-Funez P., Sanchez-Garcia J., de Mena L., Zhang Y., Levites Y., Khare S., Golde T.E., Rincon-Limas D.E. (2016). Holdase activity of secreted Hsp70 masks amyloid-beta42 neurotoxicity in *Drosophila*. Proc. Natl. Acad. Sci. USA.

[54] Younan N.D., Chen K.-F., Rose R.-S., Crowther D.C., Viles J.H. (2018). Prion protein stabilizes amyloid-β (Aβ) oligomers and enhances Aβ neurotoxicity in a *Drosophila* model of Alzheimer’s disease. J. Biol. Chem..

[55] Yamasaki Y., Tsuda L., Suzuki A., Yanagisawa K. (2018). Induction of ganglioside synthesis in *Drosophila* brain accelerates assembly of amyloid β protein. Sci. Rep..

[56] Iwata N., Tsubuki S., Takaki Y., Watanabe K., Sekiguchi M., Hosoki E., Kawashima-Morishima M., Lee H.-J., Hama E., Sekine-Aizawa Y. (2000). Identification of the major Aβ 1–42-degrading catabolic pathway in brain parenchyma: Suppression leads to biochemical and pathological deposition. Nat. Med..

[57] Iijima-Ando K., Hearn S.A., Granger L., Shenton C., Gatt A., Chiang H.-C., Hakker I., Zhong Y., Iijima K. (2008). Overexpression of neprilysin reduces alzheimer amyloid-β42 (Aβ42)-induced neuron loss and intraneuronal Aβ42 deposits but causes a reduction in cAMP-responsive element-binding protein-mediated transcription, age-dependent axon pathology, and premature death in *Drosophila*. J. Biol. Chem..

[58] Qiu W.Q., Walsh D.M., Ye Z., Vekrellis K., Zhang J., Podlisny M.B., Rosner M.R., Safavi A., Hersh L.B., Selkoe D.J. (1998). Insulin-degrading enzyme regulates extracellular levels of amyloid β-protein by degradation. J. Biol. Chem..

[59] Tsuda M., Kobayashi T., Matsuo T., Aigaki T. (2010). Insulin-degrading enzyme antagonizes insulin-dependent tissue growth and Aβ-induced neurotoxicity in *Drosophila*. FEBS Lett..

[60] Shen Y., Xia Y., Meng S., Lim N.K., Wang W., Huang F. (2017). SH2B1 is involved in the accumulation of amyloid-β 42 in Alzheimer’s disease. J. Alzheimers Dis..

[61] O’Neill C. (2013). PI3-kinase/Akt/mTOR signaling: Impaired on/off switches in aging, cognitive decline and Alzheimer’s disease. Exp. Gerontol..

[62] Boland B., Kumar A., Lee S., Platt F.M., Wegiel J., Yu W.H., Nixon R.A. (2008). Autophagy induction and autophagosome clearance in neurons: Relationship to autophagic pathology in Alzheimer’s disease. J. Neurosci..

[63] Chiang H.-C., Wang L., Xie Z., Yau A., Zhong Y. (2010). PI3 kinase signaling is involved in Aβ-induced memory loss in *Drosophila*. Proc. Natl. Acad. Sci. USA.

[64] Gerenu G., Persson T., Goikolea J., Calvo-Garrido J., Loera-Valencia R., Pottmeier P., Santiago C., Poska H., Presto J., Cedazo-Minguez A. (2019). Thioredoxin-80 protects against amyloid-beta pathology through autophagic-lysosomal pathway regulation. Mol. Psychiatry.

[65] Demirev A.V., Song H.-L., Cho M.-H., Cho K., Peak J.-J., Yoo H.J., Kim D.-H., Yoon S.-Y. (2019). V232M substitution restricts a distinct O-glycosylation of PLD3 and its neuroprotective function. Neurobiol. Dis..

[66] LaFerla F.M., Green K.N., Oddo S. (2007). Intracellular amyloid-β in Alzheimer’s disease. Nat. Rev. Neurosci..

[67] Gouras G.K., Tampellini D., Takahashi R.H., Capetillo-Zarate E. (2010). Intraneuronal β-amyloid accumulation and synapse pathology in Alzheimer’s disease. Acta Neuropathol..

[68] Hong S., Ostaszewski B.L., Yang T., O’Malley T.T., Jin M., Yanagisawa K., Li S., Bartels T., Selkoe D.J. (2014). Soluble Aβ oligomers are rapidly sequestered from brain ISF in vivo and bind GM1 ganglioside on cellular membranes. Neuron.

[69] McLaurin J., Chakrabartty A. (1997). Characterization of the interactions of Alzheimer β-amyloid peptides with phospholipid membranes. Eur. J. Biochem..

[70] McIntire L.B.J., Berman D.E., Myaeng J., Staniszewski A., Arancio O., Di Paolo G., Kim T.-W. (2012). Reduction of synaptojanin 1 ameliorates synaptic and behavioral impairments in a mouse model of Alzheimer’s disease. J. Neurosci..

[71] Zhu L., Zhong M., Zhao J., Rhee H., Caesar I., Knight E.M., Volpicelli-Daley L., Bustos V., Netzer W., Liu L. (2013). Reduction of synaptojanin 1 accelerates Aβ clearance and attenuates cognitive deterioration in an Alzheimer mouse model. J. Biol. Chem..

[72] Zhang X., Wang W.-A., Jiang L.-X., Liu H.-Y., Zhang B.-Z., Lim N., Li Q.-Y., Huang F.-D. (2017). Downregulation of RBO-PI4KIIIα facilitates Aβ42 secretion and ameliorates neural deficits in Aβ42-expressing *Drosophila*. J. Neurosci..

[73] Sun M., Zhao Y., Han M., Zhang B., Zhang X., Zhang Q., Lim N.K.-H., Wang W.-A., Huang F.-D. (2018). TTC7 and Hyccin regulate neuronal Aβ 42 accumulation and its associated neural deficits in Aβ 42-expressing *Drosophila*. J. Alzheimers Dis..

[74] Belfiori-Carrasco L.F., Marcora M.S., Bocai N.I., Ceriani M.F., Morelli L., Castaño E.M. (2017). A novel genetic screen identifies modifiers of age-dependent amyloid β toxicity in the *Drosophila* brain. Front. Aging Neurosci..

[75] Heidary G., Fortini M.E. (2001). Identification and characterization of the *Drosophila* tau homolog. Mech. Dev..

[76] Fulga T.A., Elson-Schwab I., Khurana V., Steinhilb M.L., Spires T.L., Hyman B.T., Feany M.B. (2007). Abnormal bundling and accumulation of F-actin mediates tau-induced neuronal degeneration in vivo. Nat. Cell Biol..

[77] Iijima K., Gatt A., Iijima-Ando K. (2010). Tau Ser262 phosphorylation is critical for Aβ42-induced tau toxicity in a transgenic *Drosophila* model of Alzheimer’s disease. Hum. Mol. Genet..

[78] Ando K., Maruko-Otake A., Ohtake Y., Hayashishita M., Sekiya M., Iijima K.M. (2016). Stabilization of microtubule-unbound tau via tau phosphorylation at Ser262/356 by Par-1/MARK contributes to augmentation of AD-related phosphorylation and Aβ42-induced tau toxicity. PLoS Genet..

[79] Götz J., Chen F., Van Dorpe J., Nitsch R. (2001). Formation of neurofibrillary tangles in P301L tau transgenic mice induced by Aβ42 fibrils. Science.

[80] Lewis J., Dickson D.W., Lin W.-L., Chisholm L., Corral A., Jones G., Yen S.-H., Sahara N., Skipper L., Yager D. (2001). Enhanced neurofibrillary degeneration in transgenic mice expressing mutant tau and APP. Science.

[81] Pennanen L., Götz J. (2005). Different tau epitopes define Aβ 42-mediated tau insolubility. Biochem. Biophys. Res. Commun..

[82] Oddo S., Caccamo A., Cheng D., Jouleh B., Torp R., LaFerla F.M. (2007). Genetically augmenting tau levels does not modulate the onset or progression of Aβ pathology in transgenic mice. J. Neurochem..

[83] Roberson E.D., Scearce-Levie K., Palop J.J., Yan F., Cheng I.H., Wu T., Gerstein H., Yu G.Q., Mucke L. (2007). Reducing endogenous tau ameliorates amyloid beta-induced deficits in an Alzheimer’s disease mouse model. Science.

[84] Hu S., Begum A.N., Jones M.R., Oh M.S., Beech W.K., Beech B.H., Yang F., Chen P., Ubeda O.J., Kim P.C. (2009). GSK3 inhibitors show benefits in an Alzheimer’s disease (AD) model of neurodegeneration but adverse effects in control animals. Neurobiol. Dis..

[85] Takashima A., Murayama M., Murayama O., Kohno T., Honda T., Yasutake K., Nihonmatsu N., Mercken M., Yamaguchi H., Sugihara S. (1998). Presenilin 1 associates with glycogen synthase kinase-3beta and its substrate tau. Proc. Natl. Acad. Sci. USA.

[86] Song M.S., Rauw G., Baker G.B., Kar S. (2008). Memantine protects rat cortical cultured neurons against beta-amyloid-induced toxicity by attenuating tau phosphorylation. Eur. J. Neurosci..

[87] Yang T., Knowles J.K., Lu Q., Zhang H., Arancio O., Moore L.A., Chang T., Wang Q., Andreasson K., Rajadas J. (2008). Small molecule, non-peptide p75 ligands inhibit Abeta-induced neurodegeneration and synaptic impairment. PLoS ONE.

[88] Noh M.Y., Koh S.H., Kim Y., Kim H.Y., Cho G.W., Kim S.H. (2009). Neuroprotective effects of donepezil through inhibition of GSK-3 activity in amyloid-beta-induced neuronal cell death. J. Neurochem..

[89] Drewes G., Trinczek B., Illenberger S., Biernat J., Schmitt-Ulms G., Meyer H.E., Mandelkow E.M., Mandelkow E. (1995). Microtubule-associated protein/microtubule affinity-regulating kinase (p110mark). A novel protein kinase that regulates tau-microtubule interactions and dynamic instability by phosphorylation at the Alzheimer-specific site serine 262. J. Biol. Chem..

[90] Drewes G., Ebneth A., Preuss U., Mandelkow E.M., Mandelkow E. (1997). MARK, a novel family of protein kinases that phosphorylate microtubule-associated proteins and trigger microtubule disruption. Cell.

[91] Hasegawa M., Morishima-Kawashima M., Takio K., Suzuki M., Titani K., Ihara Y. (1992). Protein sequence and mass spectrometric analyses of tau in the Alzheimer’s disease brain. J. Biol. Chem..

[92] Hanger D.P., Brion J.P., Gallo J.M., Cairns N.J., Luthert P.J., Anderton B.H. (1991). Tau in Alzheimer’s disease and Down’s syndrome is insoluble and abnormally phosphorylated. Biochem. J..

[93] Hanger D.P., Betts J.C., Loviny T.L., Blackstock W.P., Anderton B.H. (1998). New phosphorylation sites identified in hyperphosphorylated tau (paired helical filament-tau) from Alzheimer’s disease brain using nanoelectrospray mass spectrometry. J. Neurochem..

[94] Morishima-Kawashima M., Hasegawa M., Takio K., Suzuki M., Yoshida H., Titani K., Ihara Y. (1995). Proline-directed and non-proline-directed phosphorylation of PHF-tau. J. Biol. Chem..

[95] Sperber B.R., Leight S., Goedert M., Lee V.M. (1995). Glycogen synthase kinase-3 beta phosphorylates tau protein at multiple sites in intact cells. Neurosci. Lett..

[96] Singh T.J., Haque N., Grundke-Iqbal I., Iqbal K. (1995). Rapid Alzheimer-like phosphorylation of tau by the synergistic actions of non-proline-dependent protein kinases and GSK-3. FEBS Lett..

[97] Singh T.J., Wang J.Z., Novak M., Kontzekova E., Grundke-Iqbal I., Iqbal K. (1996). Calcium/calmodulin-dependent protein kinase II phosphorylates tau at Ser-262 but only partially inhibits its binding to microtubules. FEBS Lett..

[98] Sengupta A., Kabat J., Novak M., Wu Q., Grundke-Iqbal I., Iqbal K. (1998). Phosphorylation of tau at both Thr 231 and Ser 262 is required for maximal inhibition of its binding to microtubules. Arch. Biochem. Biophys..

[99] Nishimura I., Yang Y., Lu B. (2004). PAR-1 kinase plays an initiator role in a temporally ordered phosphorylation process that confers tau toxicity in *Drosophila*. Cell.

[100] Kosmidis S., Grammenoudi S., Papanikolopoulou K., Skoulakis E.M. (2010). Differential effects of Tau on the integrity and function of neurons essential for learning in *Drosophila*. J. Neurosci..

[101] Leroy K., Ando K., Laporte V., Dedecker R., Suain V., Authelet M., Heraud C., Pierrot N., Yilmaz Z., Octave J.N. (2012). Lack of tau proteins rescues neuronal cell death and decreases amyloidogenic processing of APP in APP/PS1 mice. Am. J. Pathol..

[102] Sofola O., Kerr F., Rogers I., Killick R., Augustin H., Gandy C., Allen M.J., Hardy J., Lovestone S., Partridge L. (2010). Inhibition of GSK-3 ameliorates Abeta pathology in an adult-onset *Drosophila* model of Alzheimer’s disease. PLoS Genet..

[103] Burnouf S., Gronke S., Augustin H., Dols J., Gorsky M.K., Werner J., Kerr F., Alic N., Martinez P., Partridge L. (2016). Deletion of endogenous Tau proteins is not detrimental in *Drosophila*. Sci. Rep..

[104] Klein J.A., Ackerman S.L. (2003). Oxidative stress, cell cycle, and neurodegeneration. J. Clin. Investig..

[105] Moreira P.I., Santos M.S., Oliveira C.R., Shenk J.C., Nunomura A., Smith M.A., Zhu X., Perry G. (2008). Alzheimer disease and the role of free radicals in the pathogenesis of the disease. CNS Neurol. Disord. Drug Targets.

[106] Eckert A., Schmitt K., Gotz J. (2011). Mitochondrial dysfunction—The beginning of the end in Alzheimer’s disease? Separate and synergistic modes of tau and amyloid-beta toxicity. Alzheimers Res. Ther..

[107] Rival T., Page R.M., Chandraratna D.S., Sendall T.J., Ryder E., Liu B., Lewis H., Rosahl T., Hider R., Camargo L.M. (2009). Fenton chemistry and oxidative stress mediate the toxicity of the beta-amyloid peptide in a *Drosophila* model of Alzheimer’s disease. Eur. J. Neurosci..

[108] Ott S., Dziadulewicz N., Crowther D.C. (2015). Iron is a specific cofactor for distinct oxidation- and aggregation-dependent Abeta toxicity mechanisms in a *Drosophila* model. Dis. Model. Mech..

[109] Liu B., Moloney A., Meehan S., Morris K., Thomas S.E., Serpell L.C., Hider R., Marciniak S.J., Lomas D.A., Crowther D.C. (2011). Iron promotes the toxicity of amyloid beta peptide by impeding its ordered aggregation. J. Biol. Chem..

[110] Dias-Santagata D., Fulga T.A., Duttaroy A., Feany M.B. (2007). Oxidative stress mediates tau-induced neurodegeneration in *Drosophila*. J. Clin. Investig..

[111] Lee S., Bang S.M., Hong Y.K., Lee J.H., Jeong H., Park S.H., Liu Q.F., Lee I.S., Cho K.S. (2016). The calcineurin inhibitor Sarah (Nebula) exacerbates Abeta42 phenotypes in a *Drosophila* model of Alzheimer’s disease. Dis. Model. Mech..

[112] Favrin G., Bean D.M., Bilsland E., Boyer H., Fischer B.E., Russell S., Crowther D.C., Baylis H.A., Oliver S.G., Giannakou M.E. (2013). Identification of novel modifiers of Abeta toxicity by transcriptomic analysis in the fruitfly. Sci. Rep..

[113] Muller F.L., Lustgarten M.S., Jang Y., Richardson A., Van Remmen H. (2007). Trends in oxidative aging theories. Free Radic. Biol. Med..

[114] Jung I., Kim T.Y., Kim-Ha J. (2011). Identification of *Drosophila* SOD3 and its protective role against phototoxic damage to cells. FEBS Lett..

[115] Bush A.I. (2003). The metallobiology of Alzheimer’s disease. Trends Neurosci..

[116] Lang M., Wang L., Fan Q., Xiao G., Wang X., Zhong Y., Zhou B. (2012). Genetic inhibition of solute-linked carrier 39 family transporter 1 ameliorates abeta pathology in a *Drosophila* model of Alzheimer’s disease. PLoS Genet..

[117] Lang M., Fan Q., Wang L., Zheng Y., Xiao G., Wang X., Wang W., Zhong Y., Zhou B. (2013). Inhibition of human high-affinity copper importer Ctr1 orthologous in the nervous system of *Drosophila* ameliorates Abeta42-induced Alzheimer’s disease-like symptoms. Neurobiol. Aging.

[118] Lindholm D., Wootz H., Korhonen L. (2006). ER stress and neurodegenerative diseases. Cell Death Differ..

[119] Hitomi J., Katayama T., Eguchi Y., Kudo T., Taniguchi M., Koyama Y., Manabe T., Yamagishi S., Bando Y., Imaizumi K. (2004). Involvement of caspase-4 in endoplasmic reticulum stress-induced apoptosis and Abeta-induced cell death. J. Cell Biol..

[120] Costa R.O., Ferreiro E., Cardoso S.M., Oliveira C.R., Pereira C.M. (2010). ER stress-mediated apoptotic pathway induced by Abeta peptide requires the presence of functional mitochondria. J. Alzheimers Dis..

[121] Costa R.O., Ferreiro E., Oliveira C.R., Pereira C.M. (2013). Inhibition of mitochondrial cytochrome c oxidase potentiates Abeta-induced ER stress and cell death in cortical neurons. Mol. Cell. Neurosci..

[122] Kang E.B., Kwon I.S., Koo J.H., Kim E.J., Kim C.H., Lee J., Yang C.H., Lee Y.I., Cho I.H., Cho J.Y. (2013). Treadmill exercise represses neuronal cell death and inflammation during Abeta-induced ER stress by regulating unfolded protein response in aged presenilin 2 mutant mice. Apoptosis.

[123] Ron D., Walter P. (2007). Signal integration in the endoplasmic reticulum unfolded protein response. Nat. Rev. Mol. Cell Biol..

[124] Marcora M.S., Belfiori-Carrasco L.F., Bocai N.I., Morelli L., Castano E.M. (2017). Amyloid-beta42 clearance and neuroprotection mediated by X-box binding protein 1 signaling decline with aging in the *Drosophila* brain. Neurobiol. Aging.

[125] Perry G., Roder H., Nunomura A., Takeda A., Friedlich A.L., Zhu X., Raina A.K., Holbrook N., Siedlak S.L., Harris P.L. (1999). Activation of neuronal extracellular receptor kinase (ERK) in Alzheimer disease links oxidative stress to abnormal phosphorylation. Neuroreport.

[126] Zhu X., Castellani R.J., Takeda A., Nunomura A., Atwood C.S., Perry G., Smith M.A. (2001). Differential activation of neuronal ERK, JNK/SAPK and p38 in Alzheimer disease: The ‘two hit’ hypothesis. Mech. Ageing Dev..

[127] Dineley K.T., Westerman M., Bui D., Bell K., Ashe K.H., Sweatt J.D. (2001). Beta-amyloid activates the mitogen-activated protein kinase cascade via hippocampal alpha7 nicotinic acetylcholine receptors: *In vitro* and *in vivo* mechanisms related to Alzheimer’s disease. J. Neurosci..

[128] Ma Q.L., Harris-White M.E., Ubeda O.J., Simmons M., Beech W., Lim G.P., Teter B., Frautschy S.A., Cole G.M. (2007). Evidence of Abeta- and transgene-dependent defects in ERK-CREB signaling in Alzheimer’s models. J. Neurochem..

[129] Zhu X., Lee H.-G., Raina A.K., Perry G., Smith M.A. (2002). The role of mitogen-activated protein kinase pathways in Alzheimer’s disease. Neurosignals.

[130] Cheung E.C., Slack R.S. (2004). Emerging role for ERK as a key regulator of neuronal apoptosis. Sci. STKE..

[131] Park S.H., Lee S., Hong Y.K., Hwang S., Lee J.H., Bang S.M., Kim Y.K., Koo B.S., Lee I.S., Cho K.S. (2013). Suppressive effects of SuHeXiang Wan on amyloid-beta42-induced extracellular signal-regulated kinase hyperactivation and glial cell proliferation in a transgenic *Drosophila* model of Alzheimer’s disease. Biol. Pharm. Bull..

[132] Xia Z., Dickens M., Raingeaud J., Davis R.J., Greenberg M.E. (1995). Opposing effects of ERK and JNK-p38 MAP kinases on apoptosis. Science.

[133] Cobb M.H. (1999). MAP kinase pathways. Prog. Biophys. Mol. Biol..

[134] Liu Q.F., Jeong H., Lee J.H., Hong Y.K., Oh Y., Kim Y.M., Suh Y.S., Bang S., Yun H.S., Lee K. (2016). Coriandrum sativum suppresses Abeta42-Induced ROS increases, glial cell proliferation, and ERK activation. Am. J. Chin. Med..

[135] Liu Q.F., Jeon Y., Sung Y.W., Lee J.H., Jeong H., Kim Y.M., Yun H.S., Chin Y.W., Jeon S., Cho K.S. (2018). Nardostachys jatamansi ethanol extract ameliorates Abeta42 cytotoxicity. Biol. Pharm. Bull..

[136] Rahn T., Leippe M., Roeder T., Fedders H. (2013). EGFR signaling in the brain is necessary for olfactory learning in *Drosophila* larvae. Learn. Mem..

[137] Wang L., Chiang H.C., Wu W., Liang B., Xie Z., Yao X., Ma W., Du S., Zhong Y. (2012). Epidermal growth factor receptor is a preferred target for treating amyloid-beta-induced memory loss. Proc. Natl. Acad. Sci. USA.

[138] Herrup K. (2013). Post-mitotic role of the cell cycle machinery. Curr. Opin. Cell Biol..

[139] Lee H.G., Casadesus G., Zhu X., Castellani R.J., McShea A., Perry G., Petersen R.B., Bajic V., Smith M.A. (2009). Cell cycle re-entry mediated neurodegeneration and its treatment role in the pathogenesis of Alzheimer’s disease. Neurochem. Int..

[140] Crews L., Rockenstein E., Masliah E. (2010). APP transgenic modeling of Alzheimer’s disease: Mechanisms of neurodegeneration and aberrant neurogenesis. Brain Struct. Funct..

[141] Varvel N.H., Bhaskar K., Patil A.R., Pimplikar S.W., Herrup K., Lamb B.T. (2008). Abeta oligomers induce neuronal cell cycle events in Alzheimer’s disease. J. Neurosci..

[142] Seward M.E., Swanson E., Norambuena A., Reimann A., Cochran J.N., Li R., Roberson E.D., Bloom G.S. (2013). Amyloid-beta signals through tau to drive ectopic neuronal cell cycle re-entry in Alzheimer’s disease. J. Cell Sci..

[143] Kong Y., Li K., Fu T., Wan C., Zhang D., Song H., Zhang Y., Liu N., Gan Z., Yuan L. (2016). Quercetin ameliorates Abeta toxicity in *Drosophila* AD model by modulating cell cycle-related protein expression. Oncotarget.

[144] Peng F., Zhao Y., Huang X., Chen C., Sun L., Zhuang L., Xue L. (2015). Loss of Polo ameliorates APP-induced Alzheimer’s disease-like symptoms in *Drosophila*. Sci. Rep..

[145] Alberi L., Hoey S.E., Brai E., Scotti A.L., Marathe S. (2013). Notch signaling in the brain: In good and bad times. Ageing Res. Rev..

[146] Marathe S., Liu S., Brai E., Kaczarowski M., Alberi L. (2015). Notch signaling in response to excitotoxicity induces neurodegeneration via erroneous cell cycle reentry. Cell Death Differ..

[147] Kong Y., Wu J., Zhang D., Wan C., Yuan L. (2015). The Role of miR-124 in *Drosophila* Alzheimer’s Disease Model by Targeting Delta in Notch Signaling Pathway. Curr. Mol. Med..

[148] Roth K.A. (2001). Caspases, apoptosis, and Alzheimer disease: Causation, correlation, and confusion. J. Neuropathol. Exp. Neurol..

[149] Wu S.C., Cao Z.S., Chang K.M., Juang J.L. (2017). Intestinal microbial dysbiosis aggravates the progression of Alzheimer’s disease in *Drosophila*. Nat. Commun..

[150] Hong Y.K., Lee S., Park S.H., Lee J.H., Han S.Y., Kim S.T., Kim Y.K., Jeon S., Koo B.S., Cho K.S. (2012). Inhibition of JNK/dFOXO pathway and caspases rescues neurological impairments in *Drosophila* Alzheimer’s disease model. Biochem. Biophys. Res. Commun..

[151] Hawkins C.J., Yoo S.J., Peterson E.P., Wang S.L., Vernooy S.Y., Hay B.A. (2000). The *Drosophila* caspase DRONC cleaves following glutamate or aspartate and is regulated by DIAP1, HID, and GRIM. J. Biol. Chem..

[152] Meier P., Silke J., Leevers S.J., Evan G.I. (2000). The *Drosophila* caspase DRONC is regulated by DIAP1. EMBO J..

[153] Yu S.Y., Yoo S.J., Yang L., Zapata C., Srinivasan A., Hay B.A., Baker N.E. (2002). A pathway of signals regulating effector and initiator caspases in the developing *Drosophila* eye. Development.

[154] Lin R., Angelin A., Da Settimo F., Martini C., Taliani S., Zhu S., Wallace D.C. (2014). Genetic analysis of dTSPO, an outer mitochondrial membrane protein, reveals its functions in apoptosis, longevity, and Ab42-induced neurodegeneration. Aging Cell.

[155] Goedert M., Hasegawa M., Jakes R., Lawler S., Cuenda A., Cohen P. (1997). Phosphorylation of microtubule-associated protein tau by stress-activated protein kinases. FEBS Lett..

[156] Reynolds C.H., Betts J.C., Blackstock W.P., Nebreda A.R., Anderton B.H. (2000). Phosphorylation sites on tau identified by nanoelectrospray mass spectrometry: Differences *in vitro* between the mitogen-activated protein kinases ERK2, c-Jun N-terminal kinase and P38, and glycogen synthase kinase-3beta. J. Neurochem..

[157] Zhu X., Rottkamp C.A., Boux H., Takeda A., Perry G., Smith M.A. (2000). Activation of p38 kinase links tau phosphorylation, oxidative stress, and cell cycle-related events in Alzheimer disease. J. Neuropathol. Exp. Neurol..

[158] Zhu X., Raina A.K., Rottkamp C.A., Aliev G., Perry G., Boux H., Smith M.A. (2001). Activation and redistribution of c-jun N-terminal kinase/stress activated protein kinase in degenerating neurons in Alzheimer’s disease. J. Neurochem..

[159] Song Q., Feng G., Huang Z., Chen X., Chen Z., Ping Y. (2017). Aberrant axonal arborization of PDF neurons induced by Abeta42-mediated JNK activation underlies sleep disturbance in an Alzheimer’s model. Mol. Neurobiol..

[160] Hong Y.K., Park S.H., Lee S., Hwang S., Lee M.J., Kim D., Lee J.H., Han S.Y., Kim S.T., Kim Y.K. (2011). Neuroprotective effect of SuHeXiang Wan in *Drosophila* models of Alzheimer’s disease. J. Ethnopharmacol..

[161] Wang X., Ma Y., Zhao Y., Chen Y., Hu Y., Chen C., Shao Y., Xue L. (2015). APLP1 promotes dFoxO-dependent cell death in *Drosophila*. Apoptosis.

[162] Coelho D.S., Schwartz S., Merino M.M., Hauert B., Topfel B., Tieche C., Rhiner C., Moreno E. (2018). Culling less fit neurons protects against amyloid-β-induced brain damage and cognitive and motor decline. Cell Rep..

[163] Lord J., Cruchaga C. (2014). The epigenetic landscape of Alzheimer’s disease. Nat. Neurosci..

[164] Govindarajan N., Rao P., Burkhardt S., Sananbenesi F., Schlüter O.M., Bradke F., Lu J., Fischer A. (2013). Reducing HDAC6 ameliorates cognitive deficits in a mouse model for Alzheimer’s disease. EMBO Mol. Med..

[165] Ding H., Dolan P.J., Johnson G.V. (2008). Histone deacetylase 6 interacts with the microtubule-associated protein tau. J. Neurochem..

[166] Kim C., Choi H., Jung E.S., Lee W., Oh S., Jeon N.L., Mook-Jung I. (2012). HDAC6 inhibitor blocks amyloid beta-induced impairment of mitochondrial transport in hippocampal neurons. PLoS ONE.

[167] Xiong Y., Zhao K., Wu J., Xu Z., Jin S., Zhang Y.Q. (2013). HDAC6 mutations rescue human tau-induced microtubule defects in *Drosophila*. Proc. Natl. Acad. Sci. USA.

[168] Pile L., Lee F.-H., Wassarman D. (2001). The histone deacetylase inhibitor trichostatin A influences the development of *Drosophila melanogaster*. Cell. Mol. Life. Sci..

[169] Jeong M.R., Hashimoto R., Senatorov V.V., Fujimaki K., Ren M., Lee M.S., Chuang D.-M. (2003). Valproic acid, a mood stabilizer and anticonvulsant, protects rat cerebral cortical neurons from spontaneous cell death: A role of histone deacetylase inhibition. FEBS Lett..

[170] Cao X., Südhof T.C. (2001). A transcriptively active complex of APP with Fe65 and histone acetyltransferase Tip60. Science.

[171] Baek S.H., Ohgi K.A., Rose D.W., Koo E.H., Glass C.K., Rosenfeld M.G. (2002). Exchange of N-CoR corepressor and Tip60 coactivator complexes links gene expression by NF-κB and β-amyloid precursor protein. Cell.

[172] Panikker P., Xu S.-J., Zhang H., Sarthi J., Beaver M., Sheth A., Akhter S., Elefant F. (2018). Restoring Tip60 HAT/HDAC2 balance in the neurodegenerative brain relieves epigenetic transcriptional repression and reinstates cognition. J. Neurosci..

[173] Pirooznia S.K., Sarthi J., Johnson A.A., Toth M.S., Chiu K., Koduri S., Elefant F. (2012). Tip60 HAT activity mediates APP induced lethality and apoptotic cell death in the CNS of a *Drosophila* Alzheimer’s disease model. PLoS ONE.

[174] Cutler T., Sarkar A., Moran M., Steffensmeier A., Puli O.R., Mancini G., Tare M., Gogia N., Singh A. (2015). *Drosophila* eye model to study neuroprotective role of CREB binding protein (CBP) in Alzheimer’s disease. PLoS ONE.

[175] Selkoe D.J. (2002). Alzheimer’s disease is a synaptic failure. Science.

[176] Terry R.D., Masliah E., Salmon D.P., Butters N., DeTeresa R., Hill R., Hansen L.A., Katzman R. (1991). Physical basis of cognitive alterations in Alzheimer’s disease: Synapse loss is the major correlate of cognitive impairment. Ann. Neurol..

[177] Hsia A.Y., Masliah E., McConlogue L., Yu G.-Q., Tatsuno G., Hu K., Kholodenko D., Malenka R.C., Nicoll R.A., Mucke L. (1999). Plaque-independent disruption of neural circuits in Alzheimer’s disease mouse models. Proc. Natl. Acad. Sci. USA.

[178] Lue L.-F., Kuo Y.-M., Roher A.E., Brachova L., Shen Y., Sue L., Beach T., Kurth J.H., Rydel R.E., Rogers J. (1999). Soluble amyloid β peptide concentration as a predictor of synaptic change in Alzheimer’s disease. Am. J. Pathol..

[179] Koistinaho M., Ort M., Cimadevilla J.M., Vondrous R., Cordell B., Koistinaho J., Bures J., Higgins L.S. (2001). Specific spatial learning deficits become severe with age in β-amyloid precursor protein transgenic mice that harbor diffuse β-amyloid deposits but do not form plaques. Proc. Natl. Acad. Sci. USA.

[180] Kamenetz F., Tomita T., Hsieh H., Seabrook G., Borchelt D., Iwatsubo T., Sisodia S., Malinow R. (2003). APP processing and synaptic function. Neuron.

[181] Townsend M., Shankar G.M., Mehta T., Walsh D.M., Selkoe D.J. (2006). Effects of secreted oligomers of amyloid β-protein on hippocampal synaptic plasticity: A potent role for trimers. J. Physiol..

[182] Moechars D., Dewachter I., Lorent K., Reversé D., Baekelandt V., Naidu A., Tesseur I., Spittaels K., Van Den Haute C., Checler F. (1999). Early phenotypic changes in transgenic mice that overexpress different mutants of amyloid precursor protein in brain. J. Biol. Chem..

[183] Chapman P.F., White G.L., Jones M.W., Cooper-Blacketer D., Marshall V.J., Irizarry M., Younkin L., Good M.A., Bliss T., Hyman B.T. (1999). Impaired synaptic plasticity and learning in aged amyloid precursor protein transgenic mice. Nat. Neurosci..

[184] Fitzjohn S.M., Morton R.A., Kuenzi F., Rosahl T.W., Shearman M., Lewis H., Smith D., Reynolds D.S., Davies C.H., Collingridge G.L. (2001). Age-related impairment of synaptic transmission but normal long-term potentiation in transgenic mice that overexpress the human APP695SWE mutant form of amyloid precursor protein. J. Neurosci..

[185] Fang L., Duan J., Ran D., Fan Z., Yan Y., Huang N., Gu H., Zhu Y. (2012). Amyloid-β depresses excitatory cholinergic synaptic transmission in *Drosophila*. Neurosci. Bull..

[186] Iijima K., Liu H.-P., Chiang A.-S., Hearn S.A., Konsolaki M., Zhong Y. (2004). Dissecting the pathological effects of human Aβ40 and Aβ42 in *Drosophila*: A potential model for Alzheimer’s disease. Proc. Natl. Acad. Sci. USA.

[187] Klyubin I., Walsh D.M., Lemere C.A., Cullen W.K., Shankar G.M., Betts V., Spooner E.T., Jiang L., Anwyl R., Selkoe D.J. (2005). Amyloid beta protein immunotherapy neutralizes Abeta oligomers that disrupt synaptic plasticity *in vivo*. Nat. Med..

[188] Newey S.E., Velamoor V., Govek E.E., Van Aelst L. (2005). Rho GTPases, dendritic structure, and mental retardation. J. Neurobiol..

[189] Lee T., Winter C., Marticke S.S., Lee A., Luo L. (2000). Essential roles of *Drosophila* RhoA in the regulation of neuroblast proliferation and dendritic but not axonal morphogenesis. Neuron.

[190] Nakayama A.Y., Harms M.B., Luo L. (2000). Small GTPases Rac and Rho in the maintenance of dendritic spines and branches in hippocampal pyramidal neurons. J. Neurosci..

[191] Huesa G., Baltrons M.A., Gómez-Ramos P., Morán A., García A., Hidalgo J., Francés S., Santpere G., Ferrer I., Galea E. (2010). Altered distribution of RhoA in Alzheimer’s disease and AβPP overexpressing mice. J. Alzheimers Dis..

[192] Cook M., Mani P., Wentzell J.S., Kretzschmar D. (2012). Increased RhoA prenylation in the loechrig (loe) mutant leads to progressive neurodegeneration. PLoS ONE.

[193] Wu W., Du S., Shi W., Liu Y., Hu Y., Xie Z., Yao X., Liu Z., Ma W., Xu L. (2019). Inhibition of Rac1-dependent forgetting alleviates memory deficits in animal models of Alzheimer’s disease. Protein Cell..

[194] Morfini G.A., Burns M., Binder L.I., Kanaan N.M., LaPointe N., Bosco D.A., Brown R.H., Brown H., Tiwari A., Hayward L. (2009). Axonal transport defects in neurodegenerative diseases. J. Neurosci..

[195] Gunawardena S., Goldstein L.S. (2001). Disruption of axonal transport and neuronal viability by amyloid precursor protein mutations in *Drosophila*. Neuron.

[196] Stokin G.B., Lillo C., Falzone T.L., Brusch R.G., Rockenstein E., Mount S.L., Raman R., Davies P., Masliah E., Williams D.S. (2005). Axonopathy and transport deficits early in the pathogenesis of Alzheimer’s disease. Science.

[197] Shaw J.L., Chang K.T. (2013). Nebula/DSCR1 upregulation delays neurodegeneration and protects against APP-induced axonal transport defects by restoring calcineurin and GSK-3β signaling. PLoS Genet..

[198] Rusu P., Jansen A., Soba P., Kirsch J., Löwer A., Merdes G., Kuan Y.H., Jung A., Beyreuther K., Kjaerulff O. (2007). Axonal accumulation of synaptic markers in APP transgenic *Drosophila* depends on the NPTY motif and is paralleled by defects in synaptic plasticity. Eur. J. Neurosci..

[199] Yao P.J., Zhu M., Pyun E.I., Brooks A.I., Therianos S., Meyers V.E., Coleman P.D. (2003). Defects in expression of genes related to synaptic vesicle traffickingin frontal cortex of Alzheimer’s disease. Neurobiol. Dis..

[200] Kittel R.J., Wichmann C., Rasse T.M., Fouquet W., Schmidt M., Schmid A., Wagh D.A., Pawlu C., Kellner R.R., Willig K.I. (2006). Bruchpilot promotes active zone assembly, Ca2+ channel clustering, and vesicle release. Science.

[201] Mhatre S.D., Satyasi V., Killen M., Paddock B.E., Moir R.D., Saunders A.J., Marenda D.R. (2014). Synaptic abnormalities in a *Drosophila* model of Alzheimer’s disease. Dis. Model. Mech..

[202] Huang J.-K., Ma P.-L., Ji S.-Y., Zhao X.-L., Tan J.-X., Sun X.-J., Huang F.-D. (2013). Age-dependent alterations in the presynaptic active zone in a *Drosophila* model of Alzheimer’s disease. Neurobiol. Dis..

[203] Thomas U., Ebitsch S., Gorczyca M., Koh Y., Hough C., Woods D., Gundelfinger E., Budnik V. (2000). Synaptic targeting and localization of discs-large is a stepwise process controlled by different domains of the protein. Curr. Biol..

[204] Chen K., Featherstone D.E. (2005). Discs-large (DLG) is clustered by presynaptic innervation and regulates postsynaptic glutamate receptor subunit composition in *Drosophila*. BMC Biol..

[205] Furotani K., Kamimura K., Yajima T., Nakayama M., Enomoto R., Tamura T., Okazawa H., Sone M. (2018). Suppression of the synaptic localization of a subset of proteins including APP partially ameliorates phenotypes of the *Drosophila* Alzheimer’s disease model. PLoS ONE.

[206] Verri M., Pastoris O., Dossena M., Aquilani R., Guerriero F., Cuzzoni G., Venturini L., Ricevuti G., Bongiorno A.I. (2012). Mitochondrial alterations, oxidative stress and neuroinflammation in Alzheimer’s disease. Int. J. Immunopathol. Pharmacol..

[207] Lv F., Yang X., Cui C., Su C. (2017). Exogenous expression of Drp1 plays neuroprotective roles in the Alzheimer’s disease in the Abeta42 transgenic *Drosophila* model. PLoS ONE.

[208] Verstreken P., Ly C.V., Venken K.J., Koh T.-W., Zhou Y., Bellen H.J. (2005). Synaptic mitochondria are critical for mobilization of reserve pool vesicles at *Drosophila* neuromuscular junctions. Neuron.

[209] Iijima-Ando K., Hearn S.A., Shenton C., Gatt A., Zhao L., Iijima K. (2009). Mitochondrial mislocalization underlies Aβ42-induced neuronal dysfunction in a *Drosophila* model of Alzheimer’s disease. PLoS ONE.

[210] Akiyama H., Barger S., Barnum S., Bradt B., Bauer J., Cole G.M., Cooper N.R., Eikelenboom P., Emmerling M., Fiebich B.L. (2000). Inflammation and Alzheimer’s disease. Neurobiol. Aging.

[211] Zhang B., Gaiteri C., Bodea L.-G., Wang Z., McElwee J., Podtelezhnikov A.A., Zhang C., Xie T., Tran L., Dobrin R. (2013). Integrated systems approach identifies genetic nodes and networks in late-onset Alzheimer’s disease. Cell.

[212] Wyss-Coray T., Mucke L. (2002). Inflammation in neurodegenerative disease—A double-edged sword. Neuron.

[213] Ransohoff R.M., Brown M.A. (2012). Innate immunity in the central nervous system. J. Clin. Investig..

[214] Petersen A.J., Rimkus S.A., Wassarman D.A. (2012). ATM kinase inhibition in glial cells activates the innate immune response and causes neurodegeneration in *Drosophila*. Proc. Natl. Acad. Sci. USA.

[215] Cao Y., Chtarbanova S., Petersen A.J., Ganetzky B. (2013). Dnr1 mutations cause neurodegeneration in *Drosophila* by activating the innate immune response in the brain. Proc. Natl. Acad. Sci. USA.

[216] Lee C.D., Landreth G.E. (2010). The role of microglia in amyloid clearance from the AD brain. J. Neural Transm..

[217] Coutinho-Budd J., Freeman M.R. (2013). Probing the enigma: Unraveling glial cell biology in invertebrates. Curr. Opin. Neurobiol..

[218] Ray A., Speese S.D., Logan M.A. (2017). Glial draper rescues Aβ toxicity in a *Drosophila* model of Alzheimer’s disease. J. Neurosci..

[219] Purice M.D., Speese S.D., Logan M.A. (2016). Delayed glial clearance of degenerating axons in aged *Drosophila* is due to reduced PI3K/Draper activity. Nat. Commun..

[220] Ulland T.K., Colonna M. (2018). TREM2—A key player in microglial biology and Alzheimer disease. Nat. Rev. Neurol..

[221] Tahara K., Kim H.-D., Jin J.-J., Maxwell J.A., Li L., Fukuchi K.-I. (2006). Role of toll-like receptor signalling in Aβ uptake and clearance. Brain.

[222] Cameron B., Landreth G.E. (2010). Inflammation, microglia, and Alzheimer’s disease. Neurobiol. Dis..

[223] Sessa G., Podini P., Mariani M., Meroni A., Spreafico R., Sinigaglia F., Colonna M., Panina P., Meldolesi J. (2004). Distribution and signaling of TREM2/DAP12, the receptor system mutated in human polycystic lipomembraneous osteodysplasia with sclerosing leukoencephalopathy dementia. Eur. J. Neurosci..

[224] Jiang T., Yu J.-T., Zhu X.-C., Tan L. (2013). TREM2 in Alzheimer’s disease. Mol. Neurobiol..

[225] Guerreiro R., Wojtas A., Bras J., Carrasquillo M., Rogaeva E., Majounie E., Cruchaga C., Sassi C., Kauwe J.S., Younkin S. (2013). TREM2 variants in Alzheimer’s disease. N. Engl. J. Med..

[226] Wang Y., Cella M., Mallinson K., Ulrich J.D., Young K.L., Robinette M.L., Gilfillan S., Krishnan G.M., Sudhakar S., Zinselmeyer B.H. (2015). TREM2 lipid sensing sustains the microglial response in an Alzheimer’s disease model. Cell.

[227] Sekiya M., Wang M., Fujisaki N., Sakakibara Y., Quan X., Ehrlich M.E., De Jager P.L., Bennett D.A., Schadt E.E., Gandy S. (2018). Integrated biology approach reveals molecular and pathological interactions among Alzheimer’s Aβ42, Tau, TREM2, and TYROBP in *Drosophila* models. Genome Med..

[228] Khush R.S., Leulier F., Lemaitre B. (2001). *Drosophila* immunity: Two paths to NF-kappaB. Trends Immunol..

[229] Richard K.L., Filali M., Préfontaine P., Rivest S. (2008). Toll-like receptor 2 acts as a natural innate immune receptor to clear amyloid β1–42 and delay the cognitive decline in a mouse model of Alzheimer’s disease. J. Neurosci..

[230] Scholtzova H., Kascsak R.J., Bates K.A., Boutajangout A., Kerr D.J., Meeker H.C., Mehta P.D., Spinner D.S., Wisniewski T. (2009). Induction of toll-like receptor 9 signaling as a method for ameliorating Alzheimer’s disease-related pathology. J. Neurosci..

[231] Walker D.G., Tang T.M., Lue L.-F. (2018). Increased expression of toll-like receptor 3, an anti-viral signaling molecule, and related genes in Alzheimer’s disease brains. Exp. Neurol..

[232] Tan L., Schedl P., Song H.-J., Garza D., Konsolaki M. (2008). The Toll→ NFκB signaling pathway mediates the neuropathological effects of the human Alzheimer’s Aβ42 polypeptide in *Drosophila*. PLoS ONE.

[233] Li Y., Sibon O., Dijkers P. (2018). Inhibition of NF-κB in astrocytes is sufficient to delay neurodegeneration induced by proteotoxicity in neurons. J. Neuroinflammation.

[234] Bertram L., Tanzi R.E. (2019). Alzheimer disease risk genes: 29 and counting. Nat. Rev. Neurol..

[235] Jansen I.E., Savage J.E., Watanabe K., Bryois J., Williams D.M., Steinberg S., Sealock J., Karlsson I.K., Hägg S., Athanasiu L. (2019). Genome-wide meta-analysis identifies new loci and functional pathways influencing Alzheimer’s disease risk. Nat. Genet..

[236] Ridge P.G., Hoyt K.B., Boehme K., Mukherjee S., Crane P.K., Haines J.L., Mayeux R., Farrer L.A., Pericak-Vance M.A., Schellenberg G.D. (2016). Assessment of the genetic variance of late-onset Alzheimer’s disease. Neurobiol. Aging.

[237] Hall A.M., Roberson E.D. (2012). Mouse models of Alzheimer’s disease. Brain Res. Bull..

[238] Cummings J.L., Morstorf T., Zhong K. (2014). Alzheimer’s disease drug-development pipeline: Few candidates, frequent failures. Alzheimers Res. Ther..

[239] Abbott A., Dolgin E. (2016). Failed Alzheimer’s trial does not kill leading theory of disease. Nature.

[240] Sabbagh M.N., Hendrix S., Harrison J.E. (2019). FDA position statement “Early Alzheimer’s disease: Developing drugs for treatment, Guidance for Industry”. Alzheimer’s Dement. Transl. Res. Clin. Interv..

[241] Wang X., Perumalsamy H., Kwon H.W., Na Y.-E., Ahn Y.-J. (2015). Effects and possible mechanisms of action of acacetin on the behavior and eye morphology of *Drosophila* models of Alzheimer’s disease. Sci. Rep..

[242] Lee B.I., Lee S., Suh Y.S., Lee J.S., Kim A.k., Kwon O.Y., Yu K., Park C.B. (2015). Photoexcited porphyrins as a strong suppressor of β-Amyloid aggregation and synaptic toxicity. Angew. Chem. Int. Ed..

[243] Lee B.I., Suh Y.S., Chung Y.J., Yu K., Park C.B. (2017). Shedding light on Alzheimer’s β-amyloidosis: Photosensitized methylene blue inhibits self-assembly of β-amyloid peptides and disintegrates their aggregates. Sci. Rep..

[244] Deshpande P., Gogia N., Singh A. (2019). Exploring the efficacy of natural products in alleviating Alzheimer’s disease. Neural. Regen. Res..

[245] Lee S., Bang S.M., Lee J.W., Cho K.S. (2014). Evaluation of traditional medicines for neurodegenerative diseases using *Drosophila* models. Evid. Based Complement. Altern. Med..

